# AMPK hyperactivation promotes dendrite retraction, synaptic loss, and neuronal dysfunction in glaucoma

**DOI:** 10.1186/s13024-021-00466-z

**Published:** 2021-06-29

**Authors:** Nicolas Belforte, Jessica Agostinone, Luis Alarcon-Martinez, Deborah Villafranca-Baughman, Florence Dotigny, Jorge L. Cueva Vargas, Adriana Di Polo

**Affiliations:** 1 Department of Neuroscience, Université de Montréal, Succursale centre-ville 6128, Montréal, Québec H3C 3J7 Canada; 2 Centre de recherche du Centre Hospitalier de l’Université de Montréal (CRCHUM), 900 Saint Denis Street, Montréal, Québec H2X 0A9 Canada

**Keywords:** Adenosine monophosphate-activated protein kinase, Metabolic stress, Mammalian target of rapamycin, Glaucoma, Neurodegeneration

## Abstract

**Background:**

The maintenance of complex dendritic arbors and synaptic transmission are processes that require a substantial amount of energy. Bioenergetic decline is a prominent feature of chronic neurodegenerative diseases, yet the signaling mechanisms that link energy stress with neuronal dysfunction are poorly understood. Recent work has implicated energy deficits in glaucoma, and retinal ganglion cell (RGC) dendritic pathology and synapse disassembly are key features of ocular hypertension damage.

**Results:**

We show that adenosine monophosphate-activated protein kinase (AMPK), a conserved energy biosensor, is strongly activated in RGC from mice with ocular hypertension and patients with primary open angle glaucoma. Our data demonstrate that AMPK triggers RGC dendrite retraction and synapse elimination. We show that the harmful effect of AMPK is exerted through inhibition of the mammalian target of rapamycin complex 1 (mTORC1). Attenuation of AMPK activity restores mTORC1 function and rescues dendrites and synaptic contacts. Strikingly, AMPK depletion promotes recovery of light-evoked retinal responses, improves axonal transport, and extends RGC survival.

**Conclusions:**

This study identifies AMPK as a critical nexus between bioenergetic decline and RGC dysfunction during pressure-induced stress, and highlights the importance of targeting energy homeostasis in glaucoma and other neurodegenerative diseases.

**Supplementary Information:**

The online version contains supplementary material available at 10.1186/s13024-021-00466-z.

## Background

A substantial amount of the energy produced in the brain is used for synaptic transmission. The predicted energy expenditure at dendrites is substantial [1], consistent with the high density of mitochondria and oxidative activity found in the dendritic compartment [2]. When mitochondria are compromised, they produce less ATP leading to energetic stress, which is likely to affect dendrites and their synapses. Mitochondrial dysfunction has been reported in a number of neurodegenerative diseases [3–6] and correlates with dendritic pathology and synapse disassembly [7–10]. A better understanding of how neurons sense and respond to metabolic stress will provide valuable insights into strategies aimed to prevent synaptic deficits and restore neuronal circuit function. However, the molecular mechanisms linking energy shortage with dendrite and synapse instability in neuropathological conditions are poorly understood.

Adenosine monophosphate-activated protein kinase (AMPK) is a highly conserved energy sensor and an important metabolic regulator [11]. AMPK is a heterotrimeric serine/threonine kinase composed of a catalytic α subunit (PRKAA1), which is essential for AMPK function through phosphorylation of its activation loop, and regulatory β and γ subunits [12]. During energy stress, intracellular ATP levels fall and AMP rises leading to AMPK activation [13]. Active AMPK restores energy homeostasis through catabolic pathways that produce ATP while inhibiting processes that consume energy. For example, AMPK activation during developmental energetic stress inhibits neuronal polarization and axonal outgrowth [14, 15] while it regulates synaptic remodeling in aging neurons [16]. Although sensing ATP is critical to restore energy, the consequence of persistent AMPK activity in neurons with compromised energy supply is unknown. Furthermore, the role of AMPK in dendritic and synaptic alterations in injured neurons is not well understood.

To address this knowledge gap, we focused on retinal ganglion cells (RGC), a population of long-projecting neurons that link the retina to the brain. The selective death of RGC is a crucial element in the pathophysiology of glaucoma, the leading cause of irreversible blindness worldwide [17]. Retraction of RGC dendrites with synapse disassembly is a key feature of ocular hypertension damage [7]. Recent work has strongly implicated energy deficits in glaucoma [18–23], and reduced AMPK correlates with anti-inflammatory responses and RGC protection [24]. However, the role of AMPK in RGC dendritic structure and connectivity is currently unknown. Our data reveal that AMPK is a critical mediator of RGC dendrite pathology and synapse elimination during ocular hypertension-induced metabolic stress. These findings highlight the importance of restoring energy homeostasis to recover neuronal function in glaucoma.

## Methods

### Experimental animals

All animal procedures were approved by the Centre de Recherche du Centre Hospitalier de l’Université de Montréal (CRCHUM) Animal Care Committee and the Canadian Council on Animal Care guidelines. Surgical procedures were carried out in female B6.Cg.Tg [Thy1-YFPH]2Jrs/J mice (Jackson Laboratory, Bar Harbor, ME) or wild-type littermate controls (3 – 4 months of age) maintained under 12 h light/12 h dark cyclic light conditions with an average in-cage illumination level of 10 lx. All experiments were performed under general anesthesia using 2% isoflurane (0.8 l/min), except for electroretinogram (ERG) recordings (see below).

### Magnetic microbead occlusion mouse glaucoma model

Unilateral elevation of intraocular pressure was induced by a single injection of magnetic microbeads into the anterior chamber of the eye as described [25]. Briefly, animals were anesthetized using isoflurane (2% isoflurane, 0.8 l/min) and a drop of tropicamide was applied on the cornea to induce pupil dilation (Mydriacyl, Alcon, Mississauga, ON). A custom-made sharpened microneedle attached to a microsyringe pump (World Precision Instruments, Sarasota, FL) was loaded with 1.5 μl of a homogenized magnetic microbead solution (diameter: 4.5 μm, 2.4 × 10^6^ beads) (Dynabeads M-450 Epoxy, Thermo Fisher Scientific, Waltham, MA). Using a micromanipulator, the tip of the microneedle was used to gently puncture the cornea and the microbeads were injected into the anterior chamber. A hand-held magnet was used to immediately attract the magnetic microbeads to the iridocorneal angle. This procedure avoided injury to ocular structures such as the lens and iris. Sham controls received an injection of sterile phosphate buffered saline (PBS). An antibiotic eye drop was applied to the operated eye (Alcon) and the animal was allowed to recover on a heat pad. Intraocular pressure was measured before and after the procedure (bi-weekly) in awake animals using a calibrated TonoLab rebound tonometer (Icare, Vantaa, Finland). A drop of proparacaine hydrochloride (0.5%, Alcon) was applied to the cornea and, holding the tonometer perpendicular to the eye surface, ten consecutive intraocular pressure readings per eye were taken and averaged. The following pre-established exclusion criteria were used in our study: i) mice with consecutive low intraocular pressure readings (< 40–50% increase over control) over the course of 1 week after microbead injection, ii) lens opacification, and iii) eye infection. Following these criteria, less than 10% of the total number of mice subjected to this procedure were excluded.

### Retinal and optic nerve immunohistochemistry

Animals were deeply anesthetized and transcardially perfused with 4% paraformaldehyde (PFA). The eyes were immediately collected and the retinas were dissected out. For flat-mount preparations, retinas were free-floated for 3 days in 2% Triton X-100 and 0.5% dimethyl sulfoxide (DMSO), followed by incubation in blocking solution (10% normal goat serum, 2% Triton X-100, 0.5% DMSO). The following primary antibodies were applied and incubated overnight at 4 °C: SMI-32 (NF-H, 10 μg/ml, Sternberger Monoclonals, Baltimore, MD), GFP (4 μg/ml, Sigma-Aldrich, Oakville, ON), brain-specific homeobox/POU domain protein 3A (Brn3a, 1 μg/ml, Santa Cruz Biotechnologies, Mississauga, ON), Myeloperoxidase (MPO, 10 μg/ml, R&D Systems, Minneapolis, NE), CD45 (0.01 μg/ml, Cell Signaling, Danvers, Massachusetts) and/or MHC class II (I-A/I-E, 2.5 μg/ml, Biolegend, San Diego, CA). Retinas were washed and incubated with secondary antibodies: anti-mouse Alexa Fluor 594 or anti-rabbit Alexa Fluor 488 (2 μg/ml, Molecular Probes, Eugene, OR). Retinal cryosections (12 μm) were prepared as previously described [26] and incubated overnight at 4 °C in the following primary antibodies: phospho-AMPK (pAMPK^Thr172^, 0.27 μg/ml, Sigma, St Louis, MO), Brn3a (1 μg/ml, Santa Cruz Biotechnologies), RNA-binding protein with multiple splicing (RBPMS, 0.25 μg/ml, PhosphoSolutions, Aurora, CO), phospho-S6 (Ser^240/244^, 0.12 μg/ml, Cell Signaling Technology, Boston, MA) or calbindin (1:200, Swant, Marly, Switzerland), followed by secondary anti-guinea pig or anti-rabbit antibodies (Alexa 594 or 488, 2 μg/ml, Molecular Probes). Quantification of active AMPK in RGC was carried out by co-localization of anti-pAMPK^Thr172^ antibody and the RGC-specific markers Brn3a or RBPMS. Optic nerve cross sections were prepared as described [27] and incubated with primary antibodies against the pan-microglia and macrophage marker Iba1 (ionized calcium-binding adapter molecule 1, 1.5 μg/ml, Wako Pure Chemicals Industries, Ltd., Osaka, Japan) or GFAP (glial fibrillary acidic protein, 1 μg/ml, Millipore, Temecula, CA) followed by incubation with the appropriate secondary antibodies. Samples were mounted and visualized with a Zeiss AxioSkop 2 Plus microscope (Carl Zeiss). Three retinal or optic nerve cross sections per eye were analyzed in 2 areas (central and peripheral) for a total of 6 output measures per mouse. Quantification of fluorescence was carried out using ImageJ from single in-focus plane images. The contour of individual RGC labeled for both pAMPK^Thr172^ and either Brn3a or RBPMS was outlined, and circularity, area, and mean fluorescence were measured along with adjacent background readings. The total corrected cellular fluorescence (TCCF) was calculated using the formula TCCF = integrated density – (area of selected cell × mean fluorescence of background readings). To subtract background fluorescence, we measured fluorescence intensity in three neighboring regions close to the region of interest (ROI), these values were averaged and then subtracted from the intensity value of the ROI analyzed.

### Reverse transcription and quantitative real time PCR (qPCR)

Total RNA was isolated from individual retinas using the RNeasy Mini kit (Qiagen Inc., Valencia, CA). cDNAs were generated from total RNA (1 μg) using the RevertAid First Strand cDNA Synthesis Kit (Thermo Fisher Scientific). Real-time PCR was performed using TaqMan primers against YFP (Catalog # Mr04097229_mr) or β-actin (Catalog # Mm02619580_g1) (Thermo Fisher Scientific). Amplification was performed using the 7900HT Fast Real-Time PCR System (Applied Biosystems, Waltham, MA) with the following cycle conditions: 95 °C for 15 s, 60 °C for 1 min, 72 °C for 1 min. Reactions were run in triplicates for each sample and the 2-ΔΔCt method was used for the calculation of relative gene expression**.**

### Dendritic arbor analysis

Dendritic arbor reconstruction and measurements were performed blind to manipulations. High-resolution images of YFP-labeled RGC dendrites were acquired using a confocal microscope (Leica Microsystems Inc.). Scans were taken at 0.5 μm intervals (1024 × 1024 pixels) with an average of 3 to 5 images per focal plane. Reconstruction of dendritic trees was carried out using the computer-aided filament tracing function of Imaris (Bitplane, South Windsor, CT). The following parameters were measured: i) total dendritic length: the sum of the length of all dendrites per neuron, ii) total dendritic field area: the area within the contour of the arbor created by a line connecting the outermost tips of the distal dendrites, iii) total number of branches: the sum of all dendritic branches per neuron, and iv) Sholl analysis: the number of dendrites that cross concentric circles at increasing distances from the soma (10 μm interval). RGC located in all retinal quadrants and eccentricities were included in our analysis.

### Analysis of synaptic markers

Mice were sacrificed by decapitation under deep anesthesia (5% isoflurane), and the eyes were immediately collected. The cornea was pierced with a 30-gauge needle and the eye was submerged in ice-cold 4% carbodiimide for 30 min. Retinal cryosections (16 μm) were generated and incubated overnight with each of the following primary antibodies at 4 °C: VGLUT1 (1:800, Synaptic System, Gottingen, Germany) and PSD95 (2 μg/ml, Abcam, Cambridge, UK). Sections were washed and incubated with secondary antibodies: anti-guinea pig and anti-mouse (Alexa 594 or 488, 2 μg/ml, Molecular Probes). Three retinal cross sections per eye were analyzed in 2 areas (central and peripheral) for a total of 6 output measures per mouse. Fluorescent labeling was visualized with a Leica SP5 confocal microscope (Leica Microsystems Inc.), and 7.5-μm-thick z-stacks were sequentially obtained at 0.13 μm intervals (1024 × 1024 pixels) with an average of three images per focal plane. Quantitative analysis of voxels, which measured the three-dimensional volume occupied by pre- and post-synaptic markers, was carried out using Imaris (ImarisColoc, Bitplane).

### Western blot analysis

Whole fresh retinas were rapidly isolated and homogenized in ice-cold lysis buffer: 150 mM NaCl, 20 mM Tris, pH 8.0, 1% NP-40, 0.5% Na deoxycholate, 0.1% SDS, 1 mM EDTA, supplemented with 2 mM NaVO3, and protease and phosphatase inhibitors. Protein samples were resolved on SDS polyacrylamide gels and transferred to nitrocellulose membranes (Bio-Rad Life Science, Mississauga, ON). Blots were incubated with each of the following antibodies: pAMPK^Thr172^ (0.027 μg/ml, Sigma), total AMPK (1:1000, Sigma), phospho-LKB1 (Ser^248^, 0.183 μg/ml, Sigma), total LKB1 (0.024 μg/ml, Sigma), or β-actin (0.5 μg/ml, Sigma-Aldrich), followed by anti-rabbit or anti-mouse peroxidase-linked secondary antibodies (0.5 μg/ml, GE Healthcare, Mississauga, ON). Blots were developed with a chemiluminescence reagent (ECL, Amersham Biosciences) followed by exposure of blots to ChemiDoc MP System (Bio-Rad Life Science). Analysis was performed using densitometric software (Bio-Rad Life Science) and three independent western blots were carried out using retinal samples from distinct experimental groups.

### Short interfering RNA (siRNA) and drug delivery

The following siRNA sequences against the protein kinase, AMP-activated, alpha 1 catalytic subunit (PRKAA1), which contains the Thr172 residue critical for AMPK activation [28], were purchased from Dharmacon (ON-TARGET Plus Smartpool, GE Dharmacon, Lafayette, CO): i) 5′- GCAGAAGUUUGUAGAGCAA-3′, ii) 5′- UCUUAUAGUUCAACCAUGA-3′, iii) 5′- ACCAGGAAGUCAUAC AAUA-3′, iv) 5′- CGAGUUGACCGGACAUAAA-3′ (sense strands). A non-targeting siRNA was used as control (siCTL, ON-TARGET Plus Smartpool, GE Dharmacon). In some experiments a non-targeting Cy3-tagged control siRNA (siCtl-Cy3) was used to visualize siRNA uptake by retinal cells (Thermo Fisher Scientific). Each siRNA pool (7 μg/μl, total volume: 2 μl) was injected into the vitreous chamber of the eye using a custom-made pulled glass microneedle (Wiretrol II capillary, Drummond Scientific Co., Broomall, PA). Under general anesthesia, the sclera was exposed and the tip of the needle inserted into the superior ocular quadrant at a 45° angle through the sclera and retina into the vitreous space. This route of administration avoided injury to the iris or lens that can promote RGC survival [29, 30]. The following compounds were administered by intraperitoneal injection: compound C (20 mg/kg, Sigma) or rapamycin (6 mg/kg, LC Laboratories, Woburn, MA) and control mice received vehicle.

### Human glaucoma specimens and processing

Following institutional research board approval, glaucoma human retina specimens and controls were obtained from the Human Eye Biobank for Research (HEBR, St. Michael’s Hospital, Toronto, ON). Inclusion criteria for surgical glaucoma specimens were a history of primary open angle glaucoma and histopathological demonstration of optic nerve head excavation, and age-matched controls were surgical specimens from deceased individuals without any eye pathology (Table 2). A total of 42 donor retinas were examined (27 glaucoma specimens and 15 age-matched controls). Paraffin retinal sections were heated for 20 min in citrate buffer (80-90 °C) followed by incubation in blocking solution (10% normal donkey serum, 1% bovine serum albumin, 0.5% Triton X- 100) for 1 h. Retinal sections were incubated with primary antibodies: pAMP^Thr172^ (0.27 μg/ml, Sigma) or RBPMS (0.25 μg/ml, PhosphoSolutions), followed by anti-rabbit Alexa Fluor 594 or anti-guinea pig Alexa Fluor 488 (2 μg/ml, Molecular Probes). Glaucoma and age-matched control retinas were processed simultaneously under identical conditions. All RGC in each retinal section were measured and three retinal sections per sample case were analyzed. The quantification of fluorescence and normalization for background fluorescence was carried out as described above.

### Assessment of blood-retinal-barrier integrity

Compound C (20 mg/kg, Sigma) or vehicle were administered by intraperitoneal injection using the same regimen as for dendritic and RGC density analysis (once a week for 2 weeks). Prior to imaging, mice were anesthetized and pupils were dilated as described above, and fluorescein (332 Da, 5%, Fluorescite, Novartis Pharma) was administered intraperitoneally. For fluorescein angiographic evaluation, retinal imaging was done 10 min after fluorescein administration using the Phoenix Micron IV System (Phoenix Research Labs, Pleasanton, CA). Mice subjected to transient retinal ischemia, a procedure known to compromise blood-retinal-barrier integrity [31], were used as positive controls. Briefly, under general anesthesia, the left optic nerve was exposed and the optic nerve dural sheath was opened longitudinally. A fine 10–0 nylon suture was carefully introduced between the sheath and the optic nerve and tied around the sheath to compress the ophthalmic vessels to block blood flow for 60 min [32]. Because the optic nerve sheath contains the ophthalmic artery, this procedure interrupts retinal and choroidal blood flow without damaging the optic nerve itself.

### Electroretinography

Animals were dark adapted prior to electroretinogram (ERG) recordings and all manipulations were carried out under dim red light. Mice were anesthetized using ketamine (20 mg/ml), xylazine (2 mg/ml), and acepromazine (0.4 mg/ml). Bilateral pupil dilation was induced by applying tropicamide on the cornea (1%, Mydriacyl, Alcon). The recording system used Burian-Allen bipolar electrodes adapted for mice. The active electrode, with a corneal contact shape, was placed on the cornea following application of a drop of hydroxypropyl methylcellulose (Isopto Tears, 0.5%, Alcon, Mississauga, ON). The reference electrode was placed behind the ears, and the ground electrode in the tail. Electrical signals generated in the retina were amplified (1000x) and filtered (band-pass filter: 1–1000 Hz) using a commercial amplifier (Power Lab, ADInstruments, Oxford, UK). The recorded signals were digitized (Power Lab, ADInstruments) and displayed on a computer. Bilateral ERG recordings were performed simultaneously from both eyes. Measurements from non-injured eyes (pre-injury) served as baseline, and contralateral eyes were used for data normalization. To measure the positive scotopic threshold response (pSTR), the retina was stimulated at light intensities ranging between 10^− 6^ and 10^− 4^ cd.s.m.^− 2^. A series of responses per flash were averaged for each light stimulus (50 recordings). The interval between light flashes was adjusted to allow for response recovery. A calibration protocol was established to ensure homogenous stimulation and recording parameters and was performed immediately prior to each experiment.

### Quantification of neuronal survival

Mice were subjected to transcardial perfusion with 4% PFA, retinas were dissected out and free-floated overnight at 4 °C in blocking solution (10% normal donkey serum, 2.5% bovine serum albumin, 2% Triton X-100) and incubated with Brn3a (0.3 μg/ml, Santa Cruz Biotechnologies) followed by secondary anti-goat Alexa Fluor 594 (2 μg/ml, Molecular Probes). Retinas were mounted with the nerve fiber layer side up, and visualized with a Zeiss Axio Observer (Carl Zeiss Canada). Brn3a-labeled or YFP-positive RGC were manually counted within three square areas at distances of 0.25, 0.625 and 1 mm from the optic nerve disc in each of the four retinal quadrants for a total of twelve retinal areas. For axon counts, animals received a transcardial injection of heparin in saline solution (10 U/ml) and sodium nitroprusside (10 mg/kg) followed by perfusion with 2% PFA and 2.5% glutaraldehyde. Optic nerves were dissected out, fixed in 2% osmium tetroxide, and embedded in epon resin. Semi-thin sections (0.7 μm) were cut on a microtome (Reichert, Vienna, Austria) and stained with 1% toluidine blue. RGC axons were manually counted in three non-overlapping areas of each optic nerve section, encompassing a total area of 3500 μm^2^ per nerve. The total area per optic nerve cross-section was measured using Northern Eclipse image analysis software (Empix Imaging, Toronto, ON), and this value was used to estimate the total number of axons per optic nerve. The measurements provided in this study represent a mathematical average of RGC soma and axonal density.

### Axonal transport measurements

The anterograde tracer cholera toxin B 1subunit (CTB) conjugated to Alexa Fluor 488 (Molecular Probes, Life Technologies, Eugene, OR) was injected intravitreally using a custom-made sharpened microneedle attached to a Hamilton syringe (1% diluted in sterile PBS, total volume: 2 μl). Animals were perfused transcardially with 4% PFA at 24 h after CTB administration, brains were removed, post-fixed and incubated overnight in 30% sucrose prior to embedding in optimal cutting temperature compound (Tissue-Tek, Miles Laboratories). Serial coronal cryosections of the entire superior colliculus from each animal were obtained using a cryostat (50 μm thickness). Seven sections per superior colliculus, from rostral to caudal, were selected using an unbiased systematic stereological sampling method as previously described [27]. Sections were photographed using the Zeiss Axio Observer fluorescent microscope with Apotome (Carl Zeiss Canada) and the area of the CTB signal in each section was measured using the Imaris Measurement Pro module (Bitplane, South Windsor, CT).

### Data and statistical analyses

Data analysis was always carried out masked by third party concealment of treatment using uniquely coded samples. Statistical analysis was performed using GraphPad Instat software (GraphPad Inc., San Diego, CA) by one-way analysis of variance (ANOVA) followed by a Tukey’s multiple comparison post hoc test, or by a Student’s *t*-test as indicated in the legends. All cohorts were evaluated with normality (Kolmogorov-Smirnov test) and variance (F-test) tests. Only one eye per mouse was used for glaucoma induction (or sham) and/or treatment. Therefore, the number of mice (N) indicated is equal to the number of eyes analyzed (1 eye per mouse). The number of RGC analyzed in each experiment is indicated as (n). Both N and n values are indicated in the tables and in the figure legends.

## Results

### Ocular hypertension promotes RGC dendrite retraction and synapse loss

**Fig. 1:**
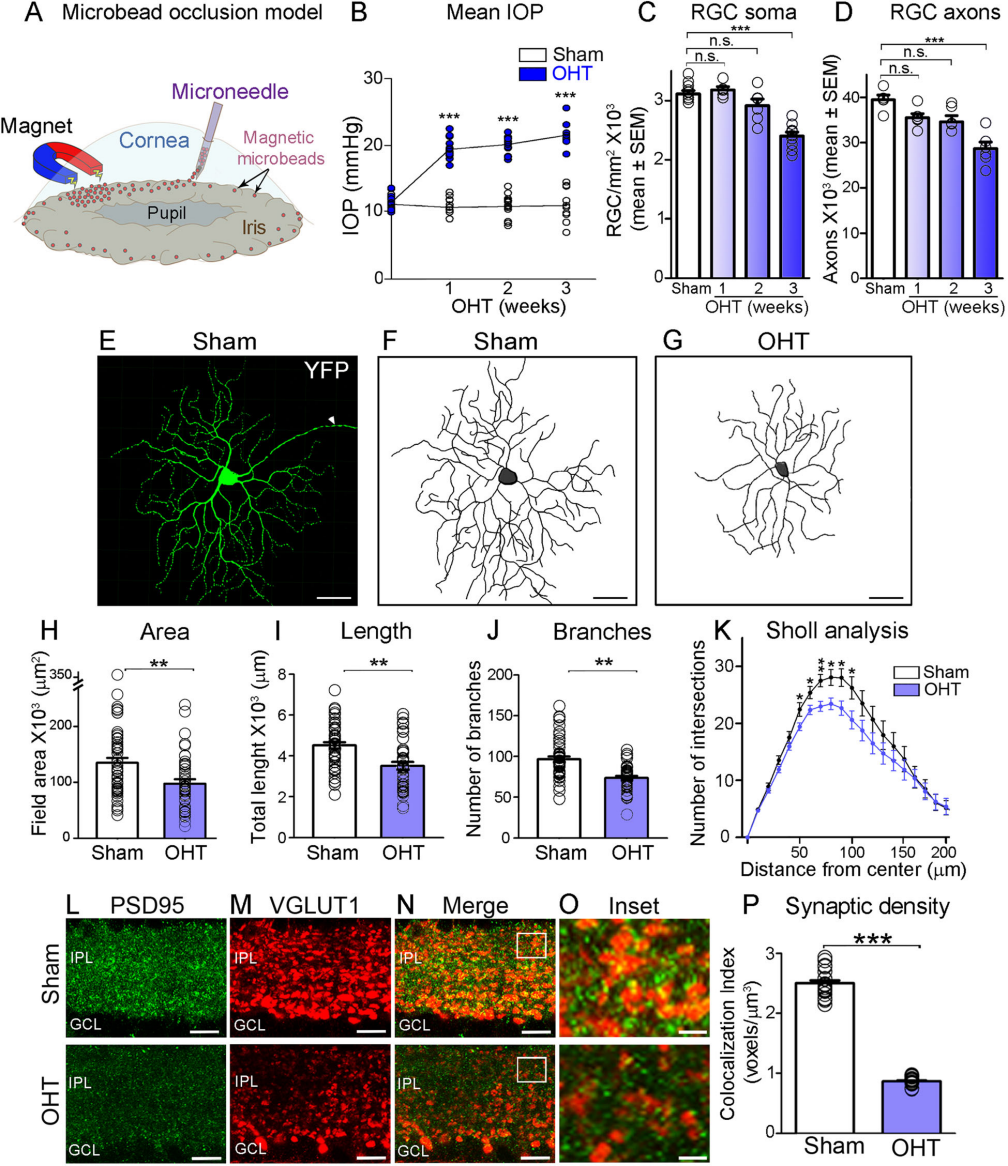
Ocular hypertension promotes early RGC dendrite retraction and synapse loss. **A** Schematic of magnetic microbeads injection into the mouse anterior chamber to induce ocular hypertension (OHT). **B** This procedure results in gradual increase of intraocular pressure (IOP) leading to RGC soma and axon loss (ANOVA with Tukey’s multiple comparison *post-hoc* test, ***p < 0.001, *N* = 10–14 mice/group). **C**, **D** Quantitative analysis demonstrates a statistically significant loss of RGC soma and axons at 3 weeks after microbeads injection (ANOVA with Tukey’s multiple comparison *post-hoc* test, n.s. = not significant, ***p < 0.001, *N* = 5–14 mice per group). (E) YFP-positive RGC with a clearly identifiable axon (arrowhead) were selected for dendritic arbor imaging and 3D reconstruction. **F**, **G** Representative examples of RGC dendritic arbors from non-injured (sham) and glaucomatous retinas at 2 weeks after induction of OHT. **H**-**K** Quantitative analysis of dendritic parameters reveals a substantial reduction in dendritic area, length, number of branches, and complexity (Sholl analysis) in OHT retinas (blue) relative to sham controls (white) (Student’s *t*-test, **p < 0.01, ***p < 0.001, *N* = 6 mice/group, *n* = 40–51 RGC/group Table 1). **L**-**N** Glutamatergic synapses are visualized in the inner plexiform layer (IPL) on retinal cross-sections using immunolabeling against PSD95 (green) and VGLUT1 (red), post- and pre-synaptic markers, respectively. OHT injury (2 weeks) induces a pronounced loss of both VGLUT1 and PSD95 expression in the IPL. **O** High magnification of inset in (N) shows VGLUT1 and PSD95 puncta in the IPL at the level of the OFF sublamina in OHT and sham retinas. **P** Quantitative analysis of pre- and postsynaptic co-localized voxels, which measured the three-dimensional volume occupied by both VGLUT1 and PSD95 in the IPL, confirms that OHT promotes a striking loss of synapses (Student’s *t-*test, ***p < 0.001, *N* = 4 mice/group). Values are expressed as the mean ± S.E.M

To establish whether dendrite pathology is a consequence of metabolic stress, we first characterized the response of RGC to ocular hypertension using a microbead occlusion model [25]. Magnetic microbeads were injected into the anterior chamber and attracted to the iridocorneal angle using a magnet to block aqueous humour outflow resulting in high intraocular pressure (Fig. 1A, B). Ocular hypertension developed as early as 1 week after the procedure, but significant RGC soma or axon loss was not detected until 3 weeks of glaucoma induction (Fig. 1C, D). Analysis of dendritic arbors and synapses was carried out at 2 weeks after microbead injection, a time when ocular hypertension was stable and prior to overt RGC soma loss (mean ± S.E.M.; sham = 3114 ± 58 RGC/mm^2^, *N* = 12 mice; ocular hypertension = 3051 ± 73 RGC/mm^2^, *N* = 15 mice, Student’s t-test, *P* = 0.5242, n.s.: not significant). Microbead occlusion was induced in mice expressing yellow fluorescent protein (YFP) under control of the Thy1 promoter (Thy1-YFPH) [33], a transgenic line that allows visualization of individual RGC dendritic trees [34]. The number of YFP-positive RGC, and the level of retinal YFP mRNA did not differ from those in non-injured retinas at 2 weeks after microbead injection (Fig. S1A, B) confirming stable reporter gene expression [35].

The antibody SMI-32, which recognizes non-phosphorylated neurofilament heavy chain, was used to identify alpha RGC characterized by strongly labeled somata and large dendritic arbors [36]. YFP-positive RGC that co-labeled with SMI-32 and had a clearly identifiable axon were selected for dendritic arbor imaging and three-dimensional reconstruction (see Methods, Fig. S1C-E). RGC located in all retinal quadrants and locations were included in our analysis and measurements were performed masked to manipulations. At 2 weeks after glaucoma induction, dendrites had visibly retracted relative to non-injured neurons from sham-injected eyes (Fig. 1E-G). Analysis of total dendritic area, length and number of branches demonstrated a reduction of 22, 28, and 24%, respectively, compared to SMI-32-positive RGC in sham controls (Fig. 1H-J, Table 1). Sholl analysis confirmed a global decrease in the number of branch intersections indicative of reduced arbor complexity (Fig. 1K).

**Table 1:** Dendritic parameters

Group	Treatment	Total dendritic lenght (μm) (mean ± SEM)	Dendritic field area (μm^**2**^) (mean ± SEM)	Number dendritic branches (mean ± SEM)	Number of animals (N)	Number of RGC (n)
Sham	–	4517 ± 154	134,899 ± 8638	97 ± 3	6	51
OHT	Vehicle	3511 ± 190	97,523 ± 7968	74 ± 3	6	40
OHT	CC	4189 ± 215	126,581 ± 11,024	92 ± 5	4	30
OHT	siCTL	2990 ± 140	83,969 ± 5709	78 ± 2	5	33
OHT	siAMPK	3910 ± 106	121,583 ± 7879	90 ± 3	5	33
OHT	siAMPK+Veh	4336 ± 122	136,330 ± 10,135	97 ± 2	4	32
OHT	siAMPK+Rapa	2454 ± 142	73,897 ± 7042	61 ± 3	4	41
Sham	Compound C	4432 ± 191	141,243 ± 9226	95 ± 5	4	28
Sham	Rapamacyn	4341 ± 201	128,375 ± 7467	94 ± 6	3	28
Sham	siAMPK	4253 ± 195	129,883 ± 10,928	94 ± 4	3	33

Next, we asked whether ocular hypertension-induced dendrite retraction correlated with loss of excitatory synapses. We examined changes in endogenous vesicular glutamate transporter 1 (VGLUT-1), a presynaptic protein expressed at bipolar ribbon synapses [37], and postsynaptic density protein 95 (PSD95) in the inner plexiform layer, where RGC dendrites are located. A pronounced decrease in the expression of both VGLUT1 and PSD95 was observed at 2 weeks after glaucoma induction relative to control retinas (Fig. 1L-O). Quantitative analysis of pre- and postsynaptic voxels, which represent the three-dimensional volume occupied by VGLUT1 and PSD95 in the inner plexiform layer, showed a 65% decrease in glaucomatous retinas compared to non-injured controls (Fig. 1P). These data confirm that RGC undergo dendritic retraction and synapse elimination in response to ocular hypertensive stress.

### Glaucoma-induced energy stress triggers AMPK activation

**Fig. 2:**
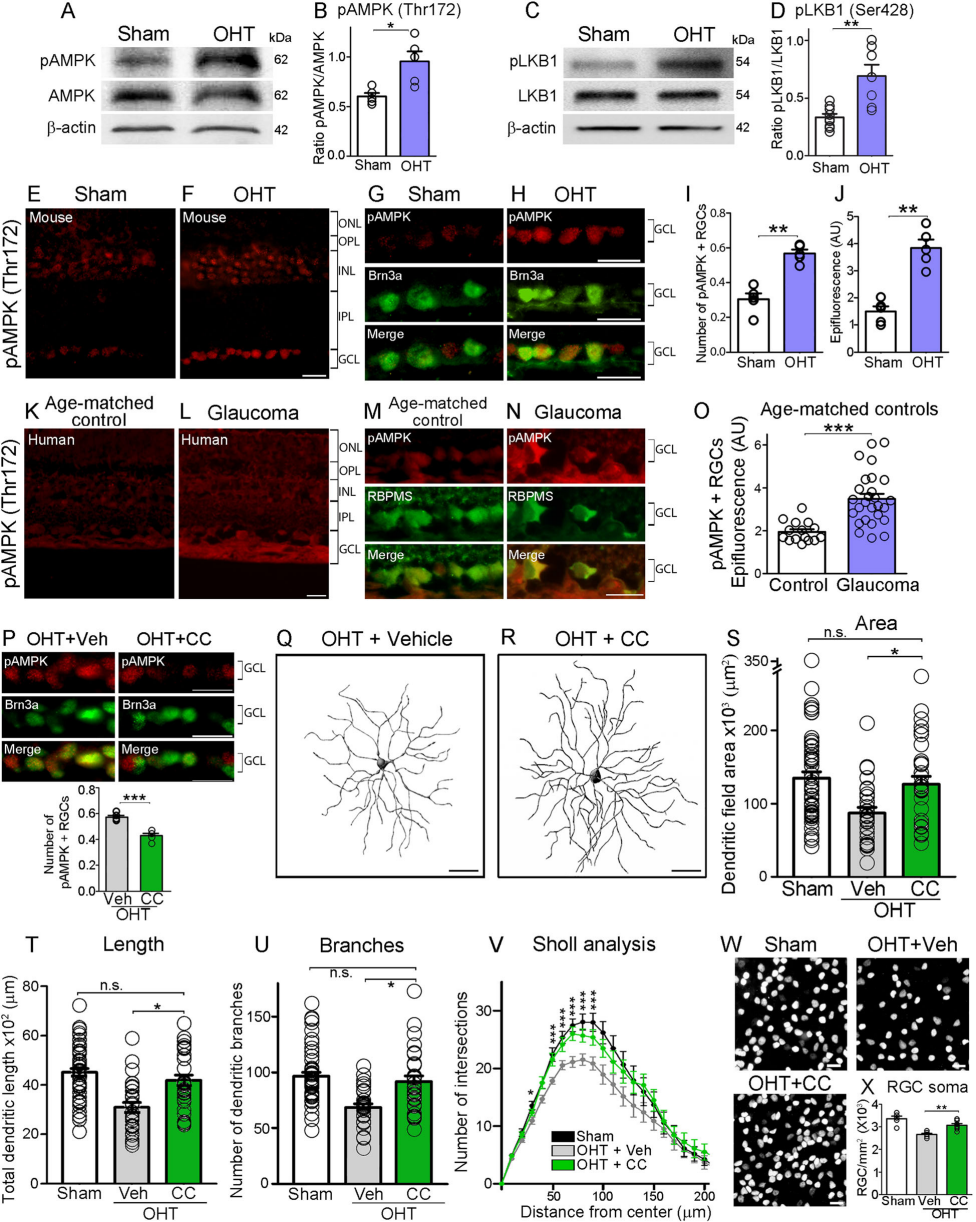
Glaucoma-induced energy stress triggers AMPK activation. **A**, **B** Western blot and densitometry analysis of retinal homogenates demonstrate a substantial increase in active AMPK (pAMPK^Thr172^), a readout of metabolic stress, as early as 1 week after induction of OHT (Student’s *t*-test, * = *p*<0.05, *N* = 5 mice/group). The lower panel is the same blot probed with an antibody against total AMPK and β-actin for normalization. **C**, **D** Western blot and densitometry analysis of retinal homogenates show increased LKB1 activity (pLKB1) in glaucomatous retinas (Student’s t-test, ** = *p*<0.01, *N* = 7–9 mice/group). The lower panel is the same blot probed with an antibody against total LKB1 and β-actin for normalization. **E**, **F** Immunohistochemical analysis of mouse retinal cross sections with an antibody against pAMPK^Thr172^, reveals robust AMPK activity in retinal cells subjected to OHT. **G**, **H** Co-labeling with antibodies against pAMPK^Thr172^ and the RGC-specific marker Brn3a demonstrates AMPK hyperactivity in RGC. **I**, **J** Quantification of the number of pAMPK^Thr172^-positive RGC as well as epifluorescence intensity per neuron confirms a significant increase in AMPK activity (Student’s t-test, ** = *p*<0.01, *N* = 5 mice/group, *n* = 50 RGC/group). **K**, **L** pAMPK^Thr172^ retinal immunostaining of primary open angle glaucoma patients and age-matched controls (Table 2) reveals increased AMPK function. **M**, **N** Co-labeling with anti-pAMPK^Thr172^ and RBPMS, a selective marker for RGC, confirms AMPK overactivation in glaucomatous RGC. **O** Quantification of epifluorescence intensity in pAMPK^Thr172^-positive RGC demonstrates a two-fold increase in AMPK activity in human glaucomatous retinas relative to age-matched controls (Student’s *t-*test, ** = *p*<0.01, glaucoma: *N* = 27 retinas/group, *n* = 100 RGC/group; controls: *N* = 15 retinas/group, *n* = 100 RGC/group). **P** Administration of compound C (CC), an inhibitor of AMPK, results a significant decrease in AMPK activity compared to vehicle-treated eyes. (Student’s t-test, *** = *p*<0.001, *N* = 5 mice/group). **Q**, **R** Representative examples of dendritic arbors from OHT retinas treated with vehicle or CC, an inhibitor of AMPK, visualized at 2 weeks after microbeads injection. **S**-**V** Quantitative analysis of dendritic parameters reveals that CC-treated neurons had longer dendrites and markedly larger and more complex arbors than vehicle-treated controls (CC: green, vehicle: grey, sham controls: white) (ANOVA with Tukey’s multiple comparison *post-hoc* test, * = *p*<0.05, *** = *p*<0.001, *N* = 4–6 mice/group, *n* = 30–40 RGC/group, Table 3). **W**, **X** Brn3a-labeled flat-mounted retinas display greater RGC soma density at 3 weeks of OHT following CC administration compared to vehicle (ANOVA with Tukey’s multiple comparison post-hoc test, ** = *p*<0.01, *N* = 5–8 mice/group). Values are expressed as the mean ± S.E.M. ONL: Outer Nuclear Layer, OPL: Outer Plexiform Layer, INL: Inner Nuclear Layer, IPL: Inner Plexiform Layer, GCL: Ganglion Cell Layer

Energy stress is characterized by low ATP and high AMP intracellular levels. When AMP binds to AMPK, it promotes allosteric changes that enhance phosphorylation of threonine 172 (Thr172) on the activation loop of the α subunit by the upstream Liver Kinase B1 (LKB1) [38, 39]. To investigate whether AMPK serves as a sensor of energy stress in glaucoma, we first performed western blot analysis of retinal homogenates using an antibody that recognizes phosphorylated AMPK on Thr172 (pAMPK^Thr172^). We observed a significant increase in retinal pAMPK^Thr172^ at 1 week after microbead injection compared to sham-injected controls (Fig. 2A, B) concomitant with enhanced LKB1 activity (Fig. 2C, D). Retinal immunohistochemistry showed pAMPK^Thr172^ increase in ocular hypertension in several cell types, consistent with the highly conserved nature of AMPK as an energy sensor and metabolic regulator [40], including RGC visualized with the cell-specific marker Brn3a (brain-specific homeobox/POU domain protein 3A) [41] (Fig. 2E-H). Quantification of active AMPK in RGC was performed by co-localization of anti-pAMPK^Thr172^ with the RGC-specific markers Brn3a or RBPMS [41, 42]. The number of pAMPK^Thr172^-positive RGC and the intensity of pAMPK^Thr172^ epifluorescence per cell confirmed enhanced AMPK activity in these neurons (Fig. 2I, J). pAMPK^Thr172^ labeling, indicative of AMPK activation, was also detected in intraretinal RGC axons and increased with glaucoma (Fig. S2).

Next, we sought to determine whether AMPK is activated in human glaucoma hence serving as an indicator of metabolic stress. For this purpose, retinal sections from 27 surgical eye specimens of patients diagnosed with primary open angle glaucoma and 15 age-matched controls were labeled for pAMPK^Thr172^ (Table 2). A substantial increase of pAMPK^Thr172^ in RGC was found in individuals with glaucoma relative to controls (Fig. 2K, L). Co-labeling of pAMPK^Thr172^ with RBPMS revealed robust AMPK activation in these neurons (Fig. 2M, N). Quantification of pAMPK^Thr172^-positive epifluorescence confirmed increased AMPK activity in RGC from glaucoma patients (Fig. 2O, *n* = 100 RGC/human retinal specimen, Student’s *t*-test, *p* < 0.001, see Methods).

**Table 2:** Patient and postmortem eye information

Patient #	Age (y)	Sex	Eye	Pathological diagnosis /cause of death	Time to fixation^a^ (h)
**Glaucoma**					
1	75	M	OD	POAG / Cancer, adenocarcinoma-liver-lung	8
2	77	M	OD	POAG / Cardiorespiratory arrest	15
3	68	F	OD	POAG / Heart disease	7
4	71	M	OD	POAG / Hypothermia	21
5	56	M	OD	POAG / Myocardial infarction	15
6	72	M	OD	POAG / Respiratory failure	11
7	71	F	OD	POAG / Respiratory failure	5
8	51	F	OS	POAG / Heart, cardiogenic shock	10
9	78	F	OD	POAG / Cancer, liver	2
10	78	F	OS	POAG / Heart disease	8
11	69	M	OD	POAG / Cerebrovascular accident	2
12	66	M	OD	POAG / Cerebrovascular accident	3
13	63	M	OD	POAG / Myocardial infarction	8
14	78	F	OD	POAG / Myocardial infarction	1
15	75	F	OD	POAG / Heart disease	5
16	71	F	OS	POAG / Respiratory failure	4
17	75	M	OS	POAG / Congestive heart failure	9
18	69	M	OD	POAG / Multiple myeloma	6
19	79	M	OD	POAG / Heart, cardiomyopathy	10
20	63	M	OD	POAG / Heart, myocardial infarction	8
21	69	M	OD	POAG / Cancer, pancreas	4
22	78	F	OS	POAG / Heart, myocardial infarction	9
23	72	F	OS	POAG / Lung disease	21
24	74	M	OD	POAG / Heart, myocardial infarction	8
25	71	F	OS	POAG / Pneumonia	6
26	68	M	OD	POAG / Cancer, lung	5
27	70	F	OD	POAG / Cancer, lung	13
**Control**					
1	74	F	OD	Cerebrovascular accident	11
2	73	M	OD	Heart disease	11
3	71	M	OD	Cerebrovascular accident	11
4	76	F	OD	Cerebrovascular accident	5
5	72	M	OD	Heart disease	6
6	59	M	OD	Myocardial infarction	12
7	72	M	OD	Cardiogenic shock	6
8	72	F	OD	Pulmonary fibrosis	5
9	70	M	OD	Cancer, lung	3
10	73	M	OD	Asthma, viral pneumonia	6
11	70	M	OD	Heart, coronary artery disease	7
12	67	F	OD	GI disease, liver failure, metastatic cancer	10
13	78	F	OD	Heart disease	17
14	77	F	OS	Heart, myocardial infarction	12
15	75	M	OS	Pulmonary embolism	12

*POAG* Primary open-angle glaucoma, *GI* Gastrointestinal, *y* Years, *h* Hours, *F* Female, *M* Male

^a^ Time from surgery or death to fixation

To test whether AMPK activation mediates dendritic pathology, we used compound C, a small molecule cell-permeable AMPK blocker [43]. Compound C or vehicle were administered by intraperitoneal injection, once a week for 2 weeks after glaucoma induction, followed by analysis of AMPK activity, RGC dendritic morphology, and RGC density. Compound C administration effectively reduced AMPK activation (Fig. 2P) and promoted robust protection of RGC dendrites preserving arbor area, process length, and complexity relative to sham-operated controls (Fig. 2Q-V). In addition, compound C effectively protected RGC soma from ocular hypertension damage (Fig. 2W, X). To rule out adverse effects of compound C including vascular leakage, we examined blood-retinal-barrier (BRB) integrity in the presence of fluorescein, a small molecular weight tracer. Retinal imaging showed no fluorescein extravasation in eyes injected with compound C or vehicle (Fig. S3A, B). In contrast, marked fluorescein extravasation was found in ischemic retinas subjected to central retinal artery ligature, a procedure known to compromise the BRB [31] and used as positive control (Fig. S3C). Consistent with this, we did not detect T cell infiltration in retinas treated with compound C (Fig. S3D), confirming that this agent does not promote vascular leakage. Collectively, our findings indicate that AMPK is markedly activated in glaucoma, a sign of metabolic stress, and that AMPK plays a role in RGC dendritic pathology.

### AMPK hyperactivation promotes dendritic retraction and synapse loss in glaucoma

**Fig. 3:**
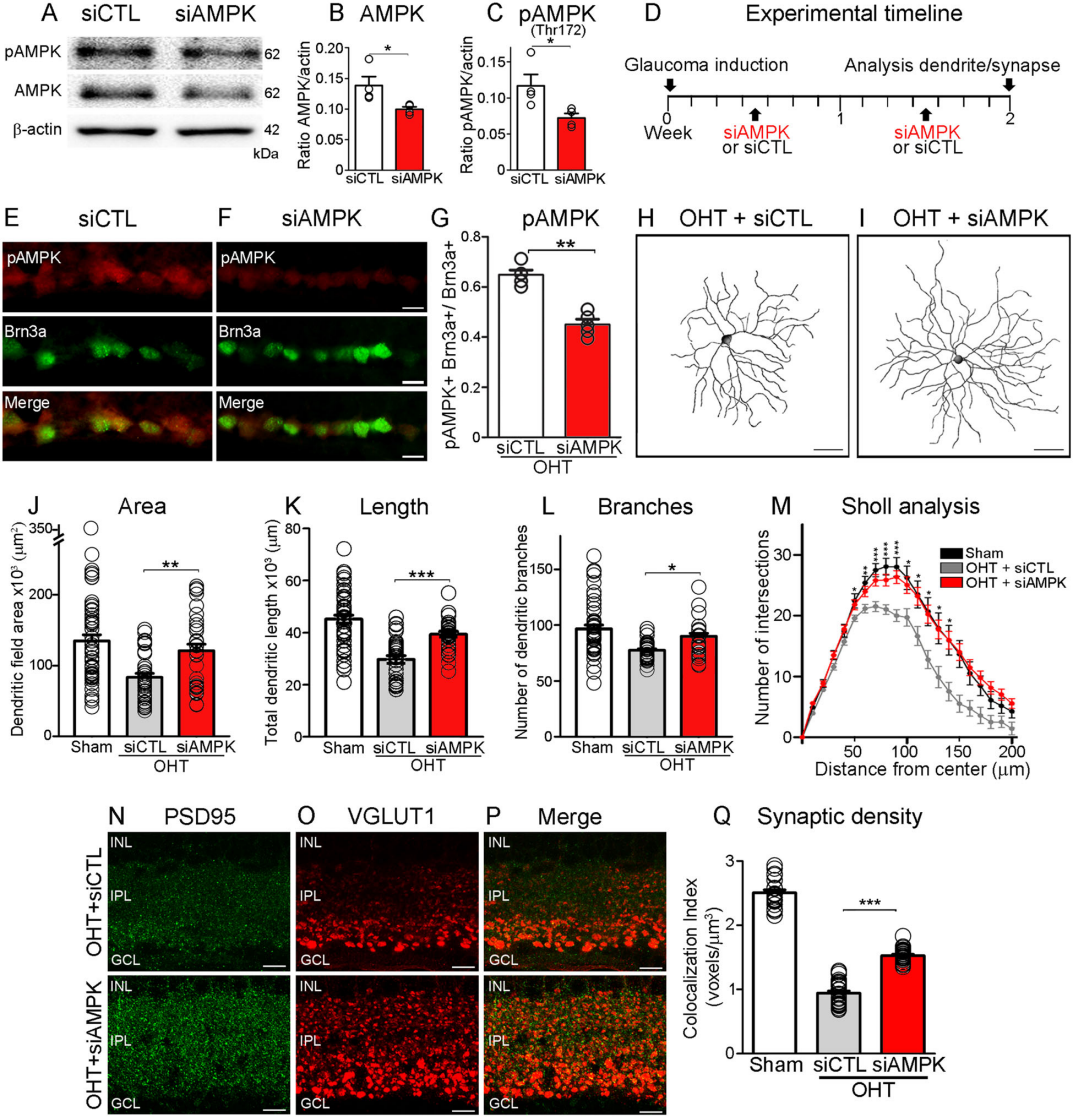
AMPK hyperactivation promotes dendritic retraction and synapse loss in glaucoma. **A**-**C** Western blot and densitometry analysis confirm that intravitreal delivery of siAMPK reduced retinal AMPK protein and its function, visualized with an antibody against pAMPK^Thr172^ (active form) (Student’s t-test, * = *p* < 0.05, *N* = 4 mice/group). The lower panel is the same blot as in the upper panels but probed with an antibody that recognizes β-actin to ensure equal protein loading. **D** siAMPK or its control siRNA (siCTL) are administered by intravitreal injection once a week, starting at 3 days after glaucoma induction. RGC dendritic and synaptic analyses are carried out at 2 weeks after injury. **E**, **F** siAMPK administration results in marked reduction of AMPK activity in RGC, visualized by co-labeling with pAMPK^Thr172^ and Brn3a on retinal cross sections. **G** Quantification of pAMPK^Thr172^- and Brn3a-positive cells confirms a significant decrease of AMPK function in glaucomatous eyes treated with siAMPK (Student’s *t*-test, **p < 0.01, *N* = 5 mice/group, *n* = 50 RGC/group). **H**, **I** siAMPK-treated RGC have longer dendrites and more elaborate arbors than control neurons treated with siCTL. **J**-**M** Quantitative analysis of dendritic parameters and Sholl analysis confirm that siAMPK-mediated AMPK knockdown significantly increased the area, length, number of branches and complexity of RGC dendrites (ANOVA with Tukey’s multiple comparison *post-hoc* test, *** = *p*<0.001, ** = *p* < 0.01, * = *p*<0.05, *N* = 5 mice/group, *n* = 30–50 RGC/group, Table 1). (N-Q) siAMPK also rescues VGLUT1 and PSD95 expression in RGC dendrites, and quantitative analysis of pre- and postsynaptic voxels confirms robust synaptic protection compared to controls (ANOVA with Tukey’s multiple comparison *post-hoc* test, *** = *p*<0.001, *N* = 5 mice/group). Values are expressed as the mean ± S.E.M. INL: Inner Nuclear Layer, IPL: Inner Plexiform Layer, GCL: Ganglion Cell Layer

To rule out potential off target effects of pharmacological AMPK inhibition [44], we selectively inhibited AMPK function using a short interfering RNA (siRNA) that targets the α catalytic subunit, which is essential for AMPK activation [39]. First, we examined whether siRNA delivered intraocularly was taken up by murine RGC. A single intravitreal injection of non-targeting Cy3-tagged control siRNA resulted in RGC labeling as early as 3 h after administration (Fig. S4A-D). The mechanism for this preferential uptake is unknown, but likely reflects the proximity of the ganglion cell layer to the vitreous chamber allowing siRNA diffusion. Next, we assessed the efficacy of a targeted siRNA against AMPK (siAMPK) to knockdown retinal AMPK protein levels and activity. Western blot analysis showed that intravitreal administration of siAMPK significantly reduced total and phosphorylated (active) AMPK, while a non-targeting control siRNA (siCTL) had no effect (Fig. 3A-C).

To assess the role of siAMPK on dendrite morphology, each siRNA (siAMPK or siCTL) was independently administered by intravitreal injection once a week for a total of 2 weeks after glaucoma induction followed by characterization of RGC morphology (Fig. 3D). Retinal immunohistochemistry confirmed a reduction in the number of pAMPK^Thr172^-positive RGC at 2 weeks of ocular hypertension in eyes treated with siAMPK relative to siCTL (Fig. 3E-G). Our data show that siAMPK-treated RGC had longer dendrites and more elaborate arbors than control neurons treated with siCTL (Fig. 3H, I). Quantitative analysis revealed that siAMPK protected dendritic field area (90%), length (86%), and number of branches (93%) while preserving arbor complexity during glaucoma-induced damage relative to siCTL (length: 66%, area: 62%, branches: 80%) (Fig. 3J-M, Table 1). siAMPK also rescued VGLUT1 and PSD95 expression in the inner plexiform layer (Fig. 3N-P), and quantitative analysis of pre- and postsynaptic voxels confirmed robust synaptic protection compared to controls (Fig. 3Q). Based on these findings, we conclude that early AMPK activation promotes RGC dendritic damage and synapse loss in glaucoma.

### AMPK knockdown rescues RGC dendrites and synapses through mTORC1 activation

**Fig. 4:**
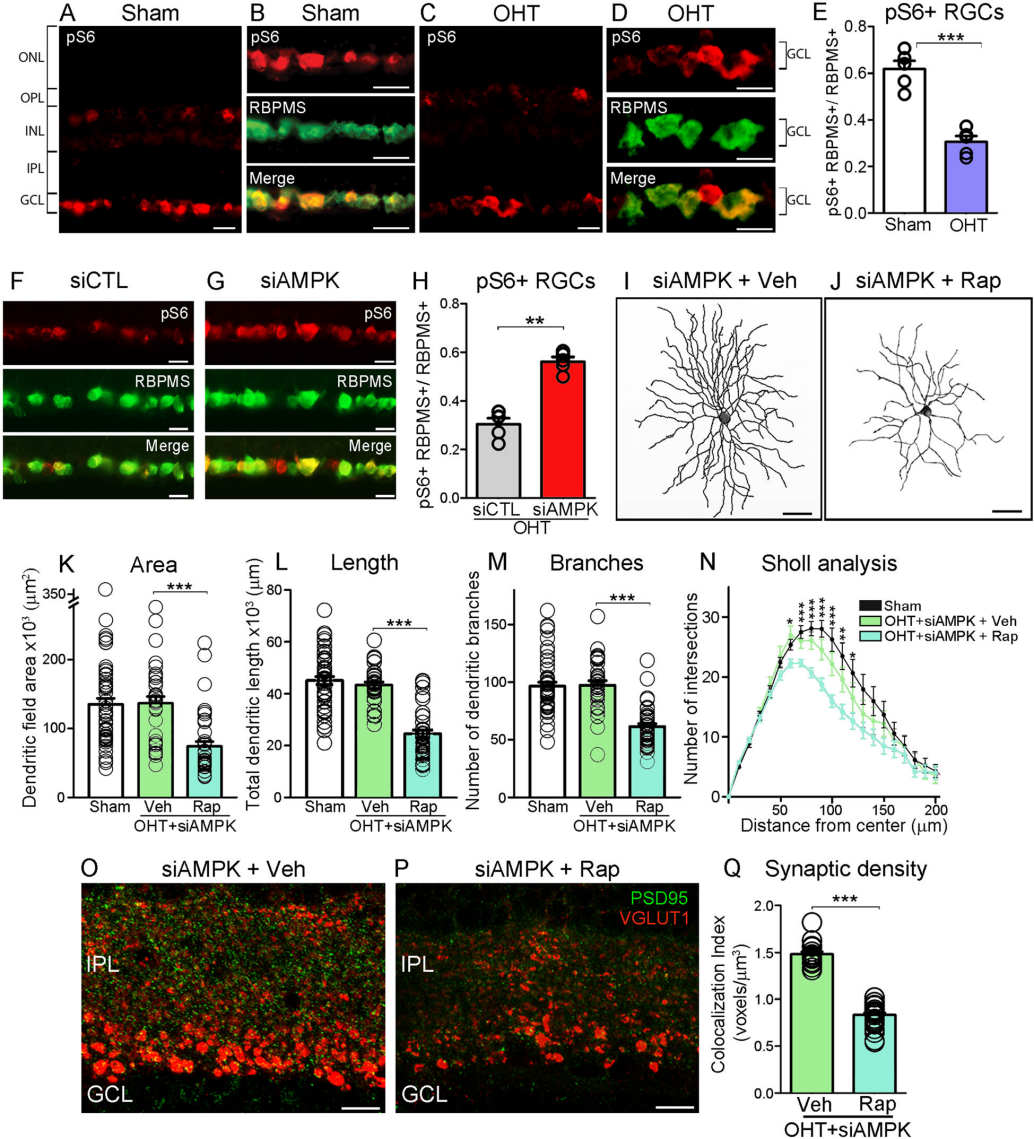
AMPK knockdown rescues RGC dendrites and synapses through mTORC1 activation. **A** Immunolabeling of non-injured sham retinas with pS6^Ser240/244^ reveals two cell populations endowed with robust constitutively active mTORC1: one located in the ganglion cell layer and another in the inner nuclear layer. **B** Co-labeling with antibodies against pS6^Ser240/244^ and RBPMS show robust mTORC1 activity in these neurons. **C**-**E** OHT markedly reduces pS6^Ser240/244^ labeling in RGC, indicating loss of mTORC1 function. **F**-**H** Administration of siAMPK in glaucomatous retinas restores mTORC1 activity in RGC relative to controls (Student’s *t*-test, ** = *p*<0.01, *N* = 5 mice/group). **I**, **J** Co-administration of siAMPK and rapamycin (Rap), an inhibitor of mTORC1, blocks the effect of siAMPK on dendritic rescue. **K**-**N** Quantification of dendritic parameters confirms a substantial reduction in area, process length, number of branches, and complexity (Sholl analysis) in rapamycin-treated retinas compared to vehicle-treated controls (ANOVA with Tukey’s multiple comparison *post-hoc* test, *** = *p*<0.001, ** = *p* < 0.01, * = *p*<0.05, *N* = 4 mice/group, *n* = 30–50 RGC/group, Table 1). **O**, **P** Rapamycin also blocks siAMPK-mediated rescue of synapses, visualized with the post- and pre-synaptic markers PSD95 and VGLUT1, respectively. **Q** Quantification of synaptic voxels confirms that siAMPK-induced synaptic protection is abolished by rapamycin, confirming that this process is mTORC1 dependent (Student’s *t*-test, ***p < 0,001, *N* = 5 mice/group). Values are expressed as the mean ± S.E.M. IPL: Inner Plexiform Layer, GCL: Ganglion Cell Layer

AMPK is a potent inhibitor of mTORC1 [45], which we previously showed is a key regulator of RGC dendritic morphology [34, 46]. To test the hypothesis that AMPK mediates dendritic pathology through inhibition of mTORC1, we first examined whether arbor retraction correlated with changes in endogenous mTORC1 function. mTORC1 activates the p70-S6 kinase leading to phosphorylation of the ribosomal protein S6 at Ser240/244 residues (pS6^Ser240/244^) thus stimulating protein translation [47]. Immunolabeling of non-injured sham retinas with pS6^Ser240/244^ revealed two cell populations endowed with robust constitutively active mTORC1: one located in the ganglion cell layer and another in the inner nuclear layer (Fig. 4A). Robust mTORC1 activity, visualized through co-labeling of pS6^Ser240/244^ and RBPMS, was found in non-injured RGC (Fig. 4B), and decreased markedly at 2 weeks of ocular hypertension (Fig. 4C-E). In the inner nuclear layer, pS6^Ser240/244^ co-localized with the calcium-binding protein calbindin, a marker of horizontal cells (Fig. S5A). In contrast to RGC, pS6^Ser240/244^ labeling in horizontal cells remained unchanged after microbead injection indicating that glaucoma-induced mTORC1 downregulation was RGC-specific (Fig. S5B). Glaucomatous retinas treated with siAMPK had substantially more pS6^Ser240/244^-positive RGC (45%) than controls (Fig. 4F-H). Co-administration of siAMPK and rapamycin, an inhibitor of mTORC1 [30], blocked the effect of siAMPK on dendritic and synaptic rescue (Fig. 4I-Q) confirming that these are mTORC1-dependent processes. Administration of siAMPK or rapamycin alone in sham-operated retinas did not elicit significant changes (Fig. S5C, D, Table 1). None of the pharmacological or siRNA reagents used in this study altered intraocular pressure (Table 3). These data identify the AMPK-mTORC1 axis as a critical regulator of RGC dendritic arbor morphology in energetically stressed neurons.

**Table 3:** Intraocular pressure elevation in glaucomatous eyes

Time after Microbead Injection	Treatment	N	Mean IOP (mm Hg) ± SEM	
			Glaucoma	Control
1 week	Sham	12	19 ± 0.5	10 ± 0.4
	Vehicle	10	20 ± 0.4	11 ± 0.9
	Compound C	9	20 ± 0.8	11 ± 0.4
	siCTL	9	19 ± 1.0	11 ± 0.5
	siAMPK	9	20 ± 0.5	11 ± 0.8
	siAMPK + Rapamycin	9	21 ± 0.4	12 ± 0.6
2 weeks	Sham	14	20 ± 0.2	11 ± 0.5
	Vehicle	10	20 ± 0.6	11 ± 0.2
	Compound C	9	24 ± 0.8	12 ± 0.8
	siCTL	9	19 ± 0.8	10 ± 0.9
	siAMPK	9	21 ± 0.5	11 ± 0.5
	siAMPK + Rapamycin	9	22 ± 0.5	11 ± 0.4
3 weeks	Sham	10	21 ± 0.5	11 ± 0.8
	siCTL	9	20 ± 0.4	11 ± 0.8
	siAMPK	9	19 ± 0.7	12 ± 0.2

### AMPK attenuation promotes RGC functional recovery and survival

**Fig. 5:**
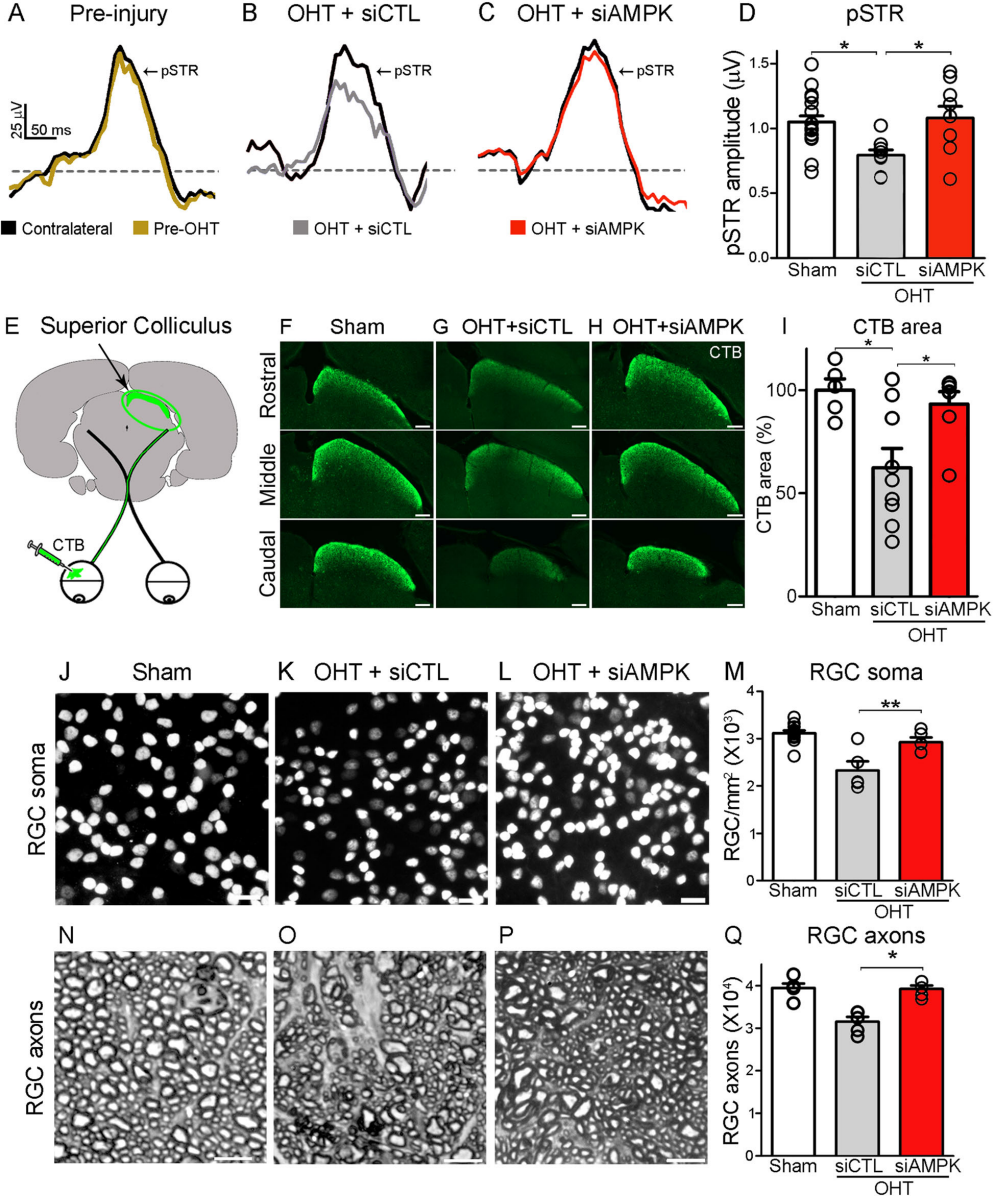
AMPK attenuation promotes RGC functional recovery and survival. **A**-**C** Representative positive scotopic threshold response (pSTR) recordings prior to induction of ocular hypertension (pre-OHT, yellow trace) or after injury and treatment with siCTL (grey trace) or siAMPK (red trace). Pre- and post-OHT recordings are normalized relative to the contralateral, non-injured eye (black traces). **D** Quantitative analysis of the pSTR amplitude demonstrates RGC function rescue in siAMPK-treated eyes relative to controls at 3 weeks after OHT induction (ANOVA with Tukey’s multiple comparison *post-hoc* test, * = *p*<0.05, *N* = 8–18 mice/group). **E** The tracer cholera toxin B subunit (CTB) conjugated to Alexa Fluor 488 is injected intravitreally and its accumulation in the contralateral superior colliculus is quantified as a readout of active anterograde axonal transport. **F**-**H** Unbiased stereological rostral-to-caudal sampling of the superior colliculi demonstrates a substantial reduction of the CTB-labeled target area in siCTL-treated mice relative to sham controls. In contrast, siAMPK-treated animals display an increase in brain CTB accumulation. **I** Quantification of the total CTB-positive area in the superior colliculus confirms a marked increase in anterograde axonal transport in siAMPK-treated mice compared to siCTL (ANOVA with Tukey’s multiple comparison *post-hoc* test, * = *p*<0.05, *N* = 5–9 mice/group). **J**-**M** Brn3a-labeled flat-mounted retinas show higher RGC soma density at 3 weeks of OHT following siAMPK treatment relative to controls (ANOVA with Tukey’s multiple comparison *post-hoc* test, * = *p*<0.05, *N* = 5–12 mice/group). **N**-**Q** siAMPK also promotes survival of RGC axons, quantified in optic nerve cross sections, compared to retinas treated with siCTL (ANOVA with Tukey’s multiple comparison *post-hoc* test, * = *p*<0.05, *N* = 5–6 mice/group). Values are expressed as the mean ± S.E.M

To assess the impact of siAMPK on RGC function, we measured the positive scotopic threshold response (pSTR), a component of the electroretinogram (ERG) that derives predominantly from RGC activity [48, 49]. We followed light responses in the same mice before and after microbead injection and administration of siAMPK or siCTL using the regimen outlined in Fig. 3D. Figure 5 shows representative pSTR recordings before (Fig. 5A, yellow trace) and after microbead injection in mice treated with siCTL (Fig. 5B, grey trace) or siAMPK (Fig. 5C, red trace) relative to contralateral eyes (black trace). The pSTR amplitude diminished in siCTL-treated glaucomatous mice while siAMPK preserved retinal responses (Fig. 5D) indicative of RGC functional recovery.

Defective anterograde transport along RGC axons is an early pathological feature of glaucoma and a sign of unhealthy neurons [50]. To investigate whether AMPK attenuation improved axonal transport, the anterograde tracer cholera toxin B subunit (CTB) was injected intraocularly and its accumulation in the contralateral superior colliculus was quantified at 3 weeks of glaucoma induction (Fig. 5E). Unbiased stereological sampling demonstrated a substantial reduction of tracer accumulation in the superior colliculus of glaucomatous mice (Fig. 5F, G), while siAMPK substantially improved CTB transport (93%) relative to controls (62%) (Fig. 5H, I).

To determine whether attenuation of AMPK activity impacted the ability of RGC to survive after injury, we used the same regimen as above and quantified RGC soma and axonal density at 3 weeks of glaucoma induction. Retinas from eyes treated with siAMPK consistently displayed higher RGC soma densities than those treated with control siRNA (Fig. 5J-L). Quantitative analysis confirmed that siAMPK promoted RGC soma survival relative to control eyes (siAMPK: 93%, siCTL: 75%) (Fig. 5M, Table 4). To rule out the possibility that changes in retinal area caused by ocular hypertension could affect RGC density, we measured the total retinal area in sham-operated and microbead-injected eyes and found no significant differences (Fig. S5E). Analysis of optic nerve cross sections showed marked protection of RGC axons in siAMPK-treated eyes compared to control nerves (siAMPK: 98%, siCTL: 78%) (Fig. 5N-Q, Table 4). This level of axonal protection together with increased pAMPK^Thr172^ in RGC axons (Fig. S2) suggest that siAMPK might act on the axonal compartment. Early changes at the optic nerve head (ONH), including infiltrating cells and reactive astrocytes, have been propose to contribute to RGC damage in glaucoma [51]. Therefore, we examined the effect of siAMPK on microglia/macrophages (Iba1) and reactive astrocytes (GFAP) at 2 weeks after glaucoma induction. Our data show that although Iba1-positive cells and GFAP immunoreactivity in the ONH increased with high intraocular pressure, siAMPK treatment did not alter this response (Fig. S6) suggesting that these events do not contribute to siAMPK-mediated RGC soma or axon protection. Collectively, these results demonstrate that attenuation of AMPK activity restores light-evoked retinal responses, improves anterograde axonal transport, and promotes robust RGC survival in glaucoma.

**Table 4:** RGC survival at 3 weeks of microbead-induced ocular hypertension (OHT)

Group	Treatment	RGCs/mm^**2**^ (mean ± SEM)	RGC axons (mean ± SEM)
Sham	None	3122 ± 57 (*n* = 12)	39,467 ± 1066 (*n* = 5)
OHT	None	2398 ± 71 (*n* = 9)	23,802 ± 1461 (*n* = 6)
OHT	siCTL	2326 ± 191 (*n* = 5)	27,420 ± 1017 (*n* = 5)
OHT	siAMPK	2924 ± 101 (*n* = 5)	39,257 ± 827 (*n* = 5)

## Discussion

RGC undergo substantial metabolic deficits in glaucoma, but how these neurons sense and respond to energetic stress is not well understood. Here, we show that RGC subjected to ocular hypertension display early signs of bioenergetic decline characterized by hyperactivation of the energy sensor AMPK. Our data show that persistent AMPK activity not only reflects metabolic strain but also exerts a marked detrimental effect on RGC structure and function. Indeed, AMPK triggered swift RGC dendritic retraction and synapse disassembly accompanied by loss of light-evoked responses and impaired axonal transport. These findings reveal that AMPK is a critical homeostatic hub which, if chronically active, contributes to RGC damage by shutting down energetically expensive processes, notably synaptic transmission and axonal transport.

Mitochondrial deficits and vascular dysregulation have been proposed to compromise energy availability in glaucoma [18–22]. Age-dependent decline in retinal nicotinamide adenine dinucleotide (NAD) was reported in an inherited glaucoma model, and administration of the NAD precursor vitamin B3 promoted neuroprotection in mice and improved retinal function in patients [23, 52]. A spectrum of mutations in mitochondrial DNA and nuclear genes encoding mitochondrial proteins have been reported in primary open angle and normal tension glaucoma patients [53–57]. We show a striking increase in AMPK activity (pAMPK^Thr172^) in RGC from both microbead-injected mice and patients affected by the disease, suggesting that AMPK is rapidly engaged in metabolically stressed RGC neurons. Of interest, a comprehensive molecular atlas of 46 RGC subtypes in the adult retina generated by single-cell RNA-seq [58] shows that *Prkaa1* expression is found in many RGC subtypes including αON sustained and transient, αOFF sustained and transient, melanopsin-positive (M1, M2 and M5), and ON-OFF direction selective RGCs (oo-DS). The widespread *Prkaa1* gene expression in most RGC classes suggests that AMPK plays an important role in the regulation of energy homeostasis beyond the αRGCs analyzed in this study.

Given the importance of AMPK to sense energy stress and mount an adaptive response, its complete inhibition can be detrimental. For example, irreversible AMPK blockade in hypoxic-ischemic brain damage was shown to exacerbate neuronal death [59]. The siRNA-based approach used here, which conferred only partial inhibition of AMPK, was surprisingly effective to protect RGC dendrites/synapses and rescue light-evoked responses. This approach is likely beneficial because it reduces the damaging consequences of AMPK overactivation while preserving its homeostatic functions. In line with this, strategies that attenuate AMPK activity rescued cognitive deficits in Alzheimer’s disease models [60, 61]. These findings suggest that the timing and duration of AMPK modulation is critical for a successful outcome in neurodegenerative diseases with a metabolic stress component.

Although AMPK has been implicated in models of brain injury and neurodegeneration [62], it is not clear how sustained AMPK activation is deleterious to neurons. We previously showed that mTORC1 is essential for RGC dendritic maintenance and regeneration [34, 46]. Here, we demonstrate that ocular hypertension-induced AMPK hyperactivity strongly inhibits mTORC1 triggering early dendrite and synapse loss in vulnerable neurons. Indeed, AMPK knockdown restores mTORC1 activity and protects dendrites in injured RGC. These data identify AMPK as a critical regulator of mTORC1-mediated maintenance of synaptic connections in energetically stressed neurons. Our results contrast with previous findings showing that the mTOR inhibitor rapamycin promoted RGC survival [63]. Rapamycin has been shown to have both beneficial and detrimental effects on metabolism, a disparity that has been largely attributed to non-specific effects depending on the time-course of administration [64, 65]. Specifically, compensatory pathways are activated in response to chronic rapamycin treatment [66, 67]. Here, we used a narrow therapeutic window to minimize off target effects (2 weeks), however sustained daily rapamycin treatment can promote neuroprotection through activation of off target pathways rather than mTORC1 inhibition [68].

## Conclusions

Our study identifies AMPK as a critical molecular link between metabolic stress and RGC dysfunction, and highlights the importance of intervention therapies that capitalize on the restoration of energy homeostasis for glaucoma.

## Supplementary Information

**Additional file 1.**

**Additional file 2: Supplementary Figure 1.** (A) The number of YFP-positive RGC does not change at 2 weeks of ocular hypertension (OHT) relative to non-injured controls (Student’s *t*-test, n.s.: not significant, *N* = 5 mice/group). (B) Real-time qPCR analysis confirms that retinal YFP gene expression does not change within 2 weeks of OHT damage (Student’s *t*-test, n.s.: not significant, *N* = 5 mice/group). (C-E) RGC co-expressing YFP and SMI-32 are selected for dendritic arbor imaging and 3D reconstruction. **Supplementary Figure 2.** Analysis of whole-mounted retinas immunolabeled with an antibody recognizing pAMPK^Thr172^ revealed a substantial increase of AMPK activity in RGC axons, visualized with SMI-32, at two weeks after glaucoma induction. Scale bars = 25 μm. **Supplementary Figure 3.** (A, B) Retinal imaging showed no fluorescein extravasation in eyes injected with compound C or vehicle. (C) In contrast, marked fluorescein extravasation was found in ischemic retinas subjected to central retinal artery ligature. (D), a procedure known to compromise the BRB [31] and used as positive control (Fig. S3C). (D, E) T cell infiltration, visualized with markers the MHCII, MPO and CD45, was not detected in retinas treated with compound C or vehicle. (F) Ischemic retinas, used as positive controls, display cells positive for MHCII, MPO and CD45. (Q) Quantification of cells expressing MHCII, MPO and CD45 confirm lack of cellular infiltrates in compound c-treated retinas. (ANOVA with Tukey’s multiple comparison post-hoc test, * = *p*<0.05, *N* = 3 mice/group). Scale bars = 10 μm. Values are expressed as the mean ± S.E.M. **Supplementary Figure 4.** (A) Intravitreal delivery of non-targeting (scrambled) Cy3-tagged control siRNA (siCTL-Cy3) results in rapid uptake by neurons in the ganglion cell layer (GCL), (B-D) identified as RBPMS-positive RGC (B, inset in A) as early as 3 h after injection. Scale bars (A) = 20 μm, (B-D) = 20 μm. OPL: Outer Plexiform Layer, INL: Inner Nuclear Layer, IPL: Inner Plexiform Layer. **Supplementary Figure 5.** (A) In the inner nuclear layer (INL), pS6^Ser240/244^ co-localizes with the calcium-binding protein calbindin, a marker of horizontal cells. (B) pS6^Ser240/244^ expression in horizontal cells remains unchanged after OHT induction (Student’s *t*-test, n.s.: not significant, *N* = 5 mice/group). (C, D) Immunohistochemical analysis of PSD95 and VGLUT1 shows no appreciable change in excitatory synapses in sham retinas treated with siAMPK and vehicle or rapamycin (ANOVA with Tukey’s multiple comparison *post-hoc* test, n.s. = not significant, *N* = 3 mice/group). (E) The total area of sham-operated versus glaucomatous retinas is similar, ruling out changes caused by ocular hypertension (Student’s *t*-test, n.s = not significant, *N* = 5–6 mice/group). Scale bars (A) = 20 μm, (C) = 10 μm. Values are expressed as the mean ± S.E.M. IPL: Inner Plexiform Layer, GCL: Ganglion Cell Layer. **Supplementary Figure 6.** (A) Immunohistochemistry of optic nerve head (ONH) cross-sections shows that although Iba1-positive cells and GFAP increase with high intraocular pressure, siAMPK treatment did not alter this response relative to control siRNA (siCTL). (B) Quantification of Iba1-positive cells and GFAP fluorescence confirmed that siAMPK does not have a significant effect on the number of Iba1-positive cells or GFAP reactivity (ANOVA with Tukey’s multiple comparison *post-hoc* test, * = *p*<0.05, ** = *p*<0.01, *N* = 3 mice/group). Scale bars = 50 μm. Values are expressed as the mean ± S.E.M.

## Data Availability

All data generated or analysed during this study are included in this published article and its supplementary information files.

## Abbreviations

AMP: Adenosine monophosphate
AMPK: Adenosine monophosphate-activated protein kinase
ATP: Adenosine triphosphate
Brn3a: Brain-specific homeobox/POU domain protein 3A
CTB: Cholera toxin B subunit
DMSO: Dimethyl sulfoxide
ERG: Electroretinogram
GFP: Green fluorescent protein
LKB1: Liver kinase B1
mTORC1: Mammalian target of rapamycin complex 1
NAD: Nicotinamide adenine dinucleotide
pAMPK^Thr172^: Phosphorylated AMPK at Thr172 residue of the α catalytic subunit
PBS: Phosphate buffered saline
PFA: Paraformaldehyde
pLKB1^Ser248^: Phosphorylated LKB1 at Ser248 residue
pS6^Ser240/244^: Phosphorylated ribosomal protein S6 at Ser240/244 residues
PSD95: Postsynaptic density protein 95
pSTR: Positive scotopic threshold response
qPCR: Quantitative real time polymerase chain reaction
RBPMS: RNA-binding protein with multiple splicing
RGC: Retinal ganglion cell
siAMPK: sirna against AMPK
siCTL: Non-targeting control siRNA
siRNA: Short interfering RNA
SMI-32: Non-phosphorylated neurofilament heavy chain
VGLUT1: Vesicular glutamate transporter 1
YFP: Yellow fluorescent protein

## Footnotes

1 Energy stress is characterized
