# Supplementary material for: AMPK hyperactivation promotes dendrite retraction, synaptic loss, and neuronal dysfunction in glaucoma
Source: Mol Neurodegener. 2021 Jun 29;16:43. doi: 10.1186/s13024-021-00466-z (PMC8243567; doi:10.1186/s13024-021-00466-z)
Supplement: Supplementary file 1 — Additional file 1. [file 13024_2021_466_MOESM1_ESM.docx]

| **Supplementary Table 1**  N=number of mice (1 eye per mouse was always used), n=number of RGC analyzed | | | | | | | | |
| --- | --- | --- | --- | --- | --- | --- | --- | --- |
|  | **Panel** | **Test** | **Cohort** | | **Mean** | **SEM** | **N** | **Normality** |
| **IOP** | 1B | ANOVA Tukey's test | Sham | 0 wks | 11.28 | 0.3288 | 11 | Yes (>0.1) |
|  |  |  |  | 1 wk | 10.81 | 0.372 | 12 |  |
|  |  |  |  | 2 wks | 10.97 | 0.4495 | 14 |  |
|  |  |  |  | 3 wks | 11.1 | 0.7847 | 10 |  |
|  |  |  | OHT | 0 wks | 11.54 | 0.3408 | 11 | Yes (>0.1) |
|  |  |  |  | 1 wk | 19.51 | 0.464 | 12 |  |
|  |  |  |  | 2 wks | 20.23 | 0.3359 | 14 |  |
|  |  |  |  | 3 wks | 21.64 | 0.5642 | 10 |  |
|  | **Panel** | **Test** | **Cohort** | **Mean** | **SEM** | **N** | | **Normality** |
| **RGC soma quantification** | 1C | ANOVA Tukey's test | Sham | 3115 | 58.68 | 12 | | Yes (>0.1) |
|  |  |  | 1 wk | 3182 | 56.63 | 5 | |  |
|  |  |  | 2 wks | 2915 | 112.8 | 6 | |  |
|  |  |  | 3 wks | 2398 | 71.2 | 9 | |  |
|  | **Panel** | **Test** | **Cohort** | **Mean** | **SEM** | **N** | | **Normality** |
| **RGC axon quantification** | 1D | ANOVA Tukey's test | Sham | 39462 | 1066 | 5 | | Yes (>0.1) |
|  |  |  | 1 wk | 35508 | 876.9 | 6 | |  |
|  |  |  | 2 wks | 34568 | 1386 | 8 | |  |
|  |  |  | 3 wks | 28632 | 1461 | 6 | |  |
|  | **Panel** | **Test** | **Cohort** | **Mean** | **SEM** | **N** | **n** | **Normality** |
| **Dendritic area** | 1H | Student's *t*-test | Sham | 134899 | 8638 | 6 | 40-51 | Yes (>0.1) |
|  |  |  | OHT | 97523 | 7968 | 6 |  |  |
|  | **Panel** | **Test** | **Cohort** | **Mean** | **SEM** | **N** | **n** | **Normality** |
| **Dendritic length** | 1I | Student's *t*-test | Sham | 4517 | 154.8 | 6 | 40-51 | Yes (>0.1) |
|  |  |  | OHT | 3511 | 190.2 | 6 |  |  |
|  | **Panel** | **Test** | **Cohort** | **Mean** | **SEM** | **N** | **n** | **Normality** |
| **Number of branches** | 1J | Student's *t*-test | Sham | 96.53 | 3.381 | 6 | 40-51 | Yes (>0.1) |
|  |  |  | OHT | 73.59 | 2.512 | 6 |  |  |
|  | **Panel** | **Test** | **Cohort** | **Mean** | **SEM** | **N** | | **Normality** |
| **Pre- and postsynaptic co-localized voxels** | 1P | Student's *t*-test | Sham | 2.504 | 0.04408 | 4 | | Yes (>0.1) |
|  |  |  | OHT | 0.8638 | 0.01543 | 4 | |  |

|  | **Panel** | **Test** | **Cohort** | **Mean** | **SEM** | **N** | | **Normality** |
| --- | --- | --- | --- | --- | --- | --- | --- | --- |
| **Densitometry analysis (pAMPK - Thr172)** | 2B | Student's *t*-test | Sham | 0.602 | 0.03441 | 5 | | Yes (>0.1) |
|  |  |  | OHT | 0.954 | 0.1009 | 5 | |  |
|  | **Panel** | **Test** | **Cohort** | **Mean** | **SEM** | **N** | | **Normality** |
| **Densitometry analysis (pLKB1 - Ser428)** | 2D | Student's *t*-test | Sham | 0.332 | 0.03062 | 9 | | Yes (>0.1) |
|  |  |  | OHT | 0.692 | 0.09597 | 7 | |  |
|  | **Panel** | **Test** | **Cohort** | **Mean** | **SEM** | **N** | **n** | **Normality** |
| **Number of pAMPK+ RGCs** | 2I | Student's *t*-test | Sham | 0.303 | 0.03384 | 5 | 50 | Yes (>0.1) |
|  |  |  | OHT | 0.567 | 0.02242 | 5 | 50 |  |
|  | **Panel** | **Test** | **Cohort** | **Mean** | **SEM** | **N** | **n** | **Normality** |
| **Epifluorescence intensity quantification** | 2J | Student's *t*-test | Sham | 1.494 | 0.1913 | 5 | 50 | Yes (>0.1) |
|  |  |  | OHT | 3.838 | 0.3104 | 5 | 50 |  |
|  | **Panel** | **Test** | **Cohort** | **Mean** | **SEM** | **N** | **n** | **Normality** |
| **Epifluorescence intensity quantification in pAMPK+ RGCs** | 2O | Student's *t*-test | Control | 1.95 | 0.1225 | 15 | 100 | Yes (>0.1) |
|  |  |  | Glaucoma | 3.488 | 0.2351 | 27 | 100 |  |
|  | **Panel** | **Test** | **Cohort** | **Mean** | **SEM** | **N** | | **Normality** |
| **Number of pAMPK+ RGCs** | 2P | Student's *t*-test | Veh (OHT) | 0.567 | 0.02242 | 5 | | Yes (>0.1) |
|  |  |  | CC (OHT) | 0.405 | 0.00625 | 5 | |  |
|  | **Panel** | **Test** | **Cohort** | **Mean** | **SEM** | **N** | **n** | **Normality** |
| **Dendritic area** | 2S | ANOVA Tukey's test | Sham | 13489 | 8637.76 | 4-6 | 30-50 | Yes (>0.1) |
|  |  |  | Veh (OHT) | 97523 | 7967.98 |  |  |  |
|  |  |  | CC (OHT) | 12658 | 11024.3 |  |  |  |
|  | **Panel** | **Test** | **Cohort** | **Mean** | **SEM** | **N** | **n** | **Normality** |
| **Dendritic length** | 2T | ANOVA Tukey's test | Sham | 4517 | 154.870 | 4-6 | 30-50 | Yes (>0.1) |
|  |  |  | Veh (OHT) | 3511 | 190.219 |  |  |  |
|  |  |  | CC (OHT) | 4189 | 214.705 |  |  |  |
|  | **Panel** | **Test** | **Cohort** | **Mean** | **SEM** | **N** | **n** | **Normality** |
| **Number of branches** | 2U | ANOVA Tukey's test | Sham | 97 | 3.36067 | 4-6 | 30-50 | Yes (>0.1) |
|  |  |  | Veh (OHT) | 74 | 2.49878 |  |  |  |
|  |  |  | CC (OHT) | 92 | 5.11207 |  |  |  |
|  | **Panel** | **Test** | **Cohort** | **Mean** | **SEM** | **N** | | **Normality** |
| **RGC soma quantification** | 2X | ANOVA Tukey's test | Sham | 3357 | 117.4 | 5 | | Yes (>0.1) |
|  |  |  | siCTL (OHT) | 2617 | 74.29 | 7 | |  |
|  |  |  | siAMPK (OHT) | 3064 | 77.21 | 7 | |  |

|  | **Panel** | **Test** | **Cohort** | **Mean** | **SEM** | **N** | | **Normality** |
| --- | --- | --- | --- | --- | --- | --- | --- | --- |
| **Densitometry analysis (AMPK)** | 3B | Student's *t*-test | siCTL | 0.1471 | 0.01425 | 5 | | Yes (>0.1) |
|  |  |  | siAMPK | 0.0984 | 0.00335 | 5 | |  |
|  | **Panel** | **Test** | **Cohort** | **Mean** | **SEM** | **N** | | **Normality** |
| **Densitometry analysis (pAMPK-Thr172)** | 3C | Student's *t*-test | siCTL | 0.128 | 0.0165 | 5 | | Yes (>0.1) |
|  |  |  | siAMPK | 0.0697 | 0.0055 | 5 | |  |
|  | **Panel** | **Test** | **Cohort** | **Mean** | **SEM** | **N** | **n** | **Normality** |
| **Quantification of pAMPK+ Brn3a+/Brn3a+ RGC Ratio** | 3G | Student's *t*-test | siCTL (OHT) | 0.6482 | 0.0188 | 5 | 50 | Yes (>0.1) |
|  |  |  | siAMPK (OHT) | 0.4501 | 0.0204 | 5 | 50 |  |
|  | **Panel** | **Test** | **Cohort** | **Mean** | **SEM** | **N** | **n** | **Normality** |
| **Dendritic area** | 3J | ANOVA Tukey's test | Sham | 134899 | 8638 | 5 | 30-50 | Yes (>0.1) |
|  |  |  | siCTL (OHT) | 83331 | 5737 | 5 |  |  |
|  |  |  | siAMPK (OHT) | 121018 | 9195 | 5 |  |  |
|  | **Panel** | **Test** | **Cohort** | **Mean** | **SEM** | **N** | **n** | **Normality** |
| **Dendritic length** | 3K | ANOVA Tukey's test | Sham | 4517 | 154.8 | 5 | 30-50 | Yes (>0.1) |
|  |  |  | siCTL (OHT) | 2974 | 149.8 | 5 |  |  |
|  |  |  | siAMPK (OHT) | 3946 | 104.9 | 5 |  |  |
|  | **Panel** | **Test** | **Cohort** | **Mean** | **SEM** | **N** | **n** | **Normality** |
| **Number of branches** | 3L | ANOVA Tukey's test | Sham | 96.53 | 3.381 | 5 | 30-50 | Yes (>0.1) |
|  |  |  | siCTL (OHT) | 77.41 | 1.464 | 5 |  |  |
|  |  |  | siAMPK (OHT) | 90.13 | 2.625 | 5 |  |  |
|  | **Panel** | **Test** | **Cohort** | **Mean** | **SEM** | **N** | | **Normality** |
| **Synaptic density** | 3Q | ANOVA Tukey's test | Sham | 2.504 | 0.0440 | 5 | | Yes (>0.1) |
|  |  |  | siCTL (OHT) | 0.9417 | 0.0324 | 5 | |  |
|  |  |  | siAMPK (OHT) | 1.526 | 0.0194 | 5 | |  |

|  | **Panel** | **Test** | **Cohort** | **Mean** | **SEM** | **N** | | **Normality** |
| --- | --- | --- | --- | --- | --- | --- | --- | --- |
| **Quantification of pS6+ RBPMS+/RBPMS+ RGC Ratio** | 4E |  | Sham | 0.6175 | 0.0352 | 5 | | Yes (>0.1) |
|  |  |  | OHT | 0.3056 | 0.0254 | 5 | |  |
|  | **Panel** | **Test** | **Cohort** | **Mean** | **SEM** | **N** | | **Normality** |
| **Quantification of pS6+ RBPMS+/RBPMS+ RGC Ratio** | 4H | Student's *t*-test | siCTL (OHT) | 0.3039 | 0.0247 | 5 | | Yes (>0.1) |
|  |  |  | siAMPK (OHT) | 0.5621 | 0.0184 | 5 | |  |
|  | **Panel** | **Test** | **Cohort** | **Mean** | **SEM** | **N** | **n** | **Normality** |
| **Dendritic area** | 4K | ANOVA Tukey's test | Sham | 13489 | 8638 | 4 | 30-50 | Yes (>0.1) |
|  |  |  | Veh (OHT+siAMPK) | 13633 | 10135 | 4 |  |  |
|  |  |  | Rap (OHT+siAMPK) | 73897 | 7042 | 4 |  |  |
|  | **Panel** | **Test** | **Cohort** | **Mean** | **SEM** | **N** | **n** | **Normality** |
| **Dendritic length** | 4L | ANOVA Tukey's test | Sham | 4517 | 154.8 | 4 | 30-50 | Yes (>0.1) |
|  |  |  | Veh (OHT+  siAMPK) | 4336 | 122.8 | 4 |  |  |
|  |  |  | Rap (OHT+  siAMPK) | 2454 | 142.8 | 4 |  |  |
|  | **Panel** | **Test** | **Cohort** | **Mean** | **SEM** | **N** | **n** | **Normality** |
| **Number of branches** | 4M | ANOVA Tukey's test | Sham | 96.53 | 3.381 | 4 | 30-50 | Yes (>0.1) |
|  |  |  | Veh (OHT+  siAMPK) | 97.3 | 4.002 | 4 |  |  |
|  |  |  | Rap (OHT+  siAMPK) | 61.24 | 2.574 | 4 |  |  |
|  | **Panel** | **Test** | **Cohort** | **Mean** | **SEM** | **N** | | **Normality** |
| **Quantification of synpatic voxels** | 4Q | Student's *t*-test | siCTL (OHT) | 1.481 | 0.02733 | 5 | | Yes (>0.1) |
|  |  |  | siAMPK (OHT) | 0.834 | 0.02191 | 5 | |  |

|  | **Panel** | **Test** | **Cohort** | **Mean** | **SEM** | **N** | **Normality** |
| --- | --- | --- | --- | --- | --- | --- | --- |
| **pSTR amplitude** | 5D | ANOVA Tukey's test | Sham | 1.052 | 0.0458 | 8-18 | Yes(>0.1) |
|  |  |  | siCTL (OHT) | 0.794 | 0.0412 |  |  |
|  |  |  | siAMPK (OHT) | 1.083 | 0.0900 |  |  |
|  | **Panel** | **Test** | **Cohort** | **Mean** | **SEM** | **N** | **Normality** |
| **CTB** | 5I | ANOVA Tukey's test | Sham | 100 | 5.5 | 5-9 | Yes(>0.1) |
|  |  |  | siCTL (OHT) | 62.29 | 9.382 |  |  |
|  |  |  | siAMPK (OHT) | 93.15 | 6.119 |  |  |
|  | **Panel** | **Test** | **Cohort** | **Mean** | **SEM** | **N** | **Normality** |
| **RGC soma quantification** | 5M | ANOVA Tukey's test | Sham | 3115 | 58.68 | 5-12 | Yes(>0.1) |
|  |  |  | siCTL (OHT) | 2326 | 191.8 |  |  |
|  |  |  | siAMPK (OHT) | 2924 | 101.9 |  |  |
|  | **Panel** | **Test** | **Cohort** | **Mean** | **SEM** | **N** | **Normality** |
| **RGC axon quantification** | 5Q | ANOVA Tukey's test | Sham | 39462 | 1066 | 5-6 | Yes(>0.1) |
|  |  |  | siCTL (OHT) | 27420 | 1017 |  |  |
|  |  |  | siAMPK (OHT) | 39257 | 827.2 |  |  |

|  | **Panel** | **Test** | **Cohort** | **Mean** | **SEM** | **N** | **Normality** |
| --- | --- | --- | --- | --- | --- | --- | --- |
| **Quantification of YFP+ RGCs per retina** | Suppl. 1A | Student's *t*-test | Sham | 55.6 | 11.74 | 5 | Yes (>0.1) |
|  |  |  | OHT | 57.8 | 12.06 | 5 |  |
|  | **Panel** | **Test** | **Cohort** | **Mean** | **SEM** | **N** | **Normality** |
| **RT qPCR analysis** | Suppl. 1B | Student's *t*-test | Sham | 1.043 | 0.1494 | 5 | Yes (>0.1) |
|  |  |  | OHT | 1.027 | 0.2162 | 5 |  |

|  | **Panel** | **Test** | **Cohort** | **Mean** | **SEM** | **N** | **Normality** |
| --- | --- | --- | --- | --- | --- | --- | --- |
| **Quantification of MHCII+ MPO+ CD45+ infiltrating cells** | Suppl. 3D | ANOVA Tukey's test | Sham | 72 | 5.196 | 3 | N/A |
|  |  |  | CC | 81 | 5.196 | 3 |  |
|  |  |  | Isch | 303 | 33 | 3 |  |

|  | **Panel** | **Test** | **Cohort** | **Mean** | **SEM** | **N** | **Normality** |
| --- | --- | --- | --- | --- | --- | --- | --- |
| **Quantification of pS6+ horizontal cells** | Suppl. 5B | Student's *t*-test | Sham | 0.8305 | 0.02368 | 5 | Yes(>0.1) |
|  |  |  | OHT | 0.8338 | 0.01852 | 5 |  |
|  | **Panel** | **Test** | **Cohort** | **Mean** | **SEM** | **N** |  |
| **Immunohistochemistry analysis of PSD95 and VGLUT1** | Suppl. 5D | ANOVA Tukey's test | Sham | 2.504 | 0.04408 | 3 | Yes(>0.1) |
|  |  |  | Veh (sham+siAMPK) | 2.514 | 0.06068 | 3 |  |
|  |  |  | Rap (sham+siAMPK) | 2.425 | 0.0555 | 3 |  |
|  | **Panel** | **Test** | **Cohort** | **Mean** | **SEM** | **N** | **Normality** |
| **Total retina area** | Suppl. 5E | Student's *t*-test | Sham | 16.94 | 0.4841 | 5 | Yes(>0.1) |
|  |  |  | OHT | 16.56 | 0.4941 | 5 |  |

|  | **Panel** | **Test** | **Cohort** | **Mean** | **SEM** | **N** | **Normality** |
| --- | --- | --- | --- | --- | --- | --- | --- |
| **Immunohistochemistry analysis of Iba1+ cells** | Suppl. 6B | Student's *t*-test | Sham | 2.333 | 0.3333 | 3 | Yes(>0.1) |
|  |  |  | siCTL (OHT) | 4.667 | 0.3333 | 3 |  |
|  |  |  | siAMPK (OHT) | 5.333 | 0.3333 | 3 |  |
|  | **Panel** | **Test** | **Cohort** | **Mean** | **SEM** | **N** | **Normality** |
| **GFAP epifluorescence intensity** | Suppl. 6B | Student's *t*-test | Sham | 0.6645 | 0.1075 | 3 | Yes(>0.1) |
|  |  |  | siCTL (OHT) | 2.265 | 0.3864 | 3 |  |
|  |  |  | siAMPK (OHT) | 2.229 | 0.3383 | 3 |  |
