# Supplementary material for: AMPK hyperactivation promotes dendrite retraction, synaptic loss, and neuronal dysfunction in glaucoma
Source: Mol Neurodegener. 2021 Jun 29;16:43. doi: 10.1186/s13024-021-00466-z (PMC8243567; doi:10.1186/s13024-021-00466-z)
Supplement: Supplementary file 2 — Additional file 2: Supplementary Figure 1. (A) The number of YFP-positive RGC does not change at 2 weeks of ocular hypertension (OHT) relative to non-injured controls (Student’s t-test, n.s.: not significant, N = 5 mice/group). (B) Real-time qPCR analysis confirms that retinal YFP gene expression does not change within 2 weeks of OHT damage (Student’s t-test, n.s.: not significant, N = 5 mice/group). (C-E) RGC co-expressing YFP and SMI-32 are selected for dendritic arbor imaging and 3D reconstruction. Supplementary Figure 2. Analysis of whole-mounted retinas immunolabeled with an antibody recognizing pAMPKThr172 revealed a substantial increase of AMPK activity in RGC axons, visualized with SMI-32, at two weeks after glaucoma induction. Scale bars = 25 μm. Supplementary Figure 3. (A, B) Retinal imaging showed no fluorescein extravasation in eyes injected with compound C or vehicle. (C) In contrast, marked fluorescein extravasation was found in ischemic retinas subjected to central retinal artery ligature. (D), a procedure known to compromise the BRB [31] and used as positive control (Fig. S3C). (D, E) T cell infiltration, visualized with markers the MHCII, MPO and CD45, was not detected in retinas treated with compound C or vehicle. (F) Ischemic retinas, used as positive controls, display cells positive for MHCII, MPO and CD45. (Q) Quantification of cells expressing MHCII, MPO and CD45 confirm lack of cellular infiltrates in compound c-treated retinas. (ANOVA with Tukey’s multiple comparison post-hoc test, * = p<0.05, N = 3 mice/group). Scale bars = 10 μm. Values are expressed as the mean ± S.E.M. Supplementary Figure 4. (A) Intravitreal delivery of non-targeting (scrambled) Cy3-tagged control siRNA (siCTL-Cy3) results in rapid uptake by neurons in the ganglion cell layer (GCL), (B-D) identified as RBPMS-positive RGC (B, inset in A) as early as 3 h after injection. Scale bars (A) = 20 μm, (B-D) = 20 μm. OPL: Outer Plexiform Layer, INL: Inner Nuclear Layer, IPL: Inner [file 13024_2021_466_MOESM2_ESM.pdf]

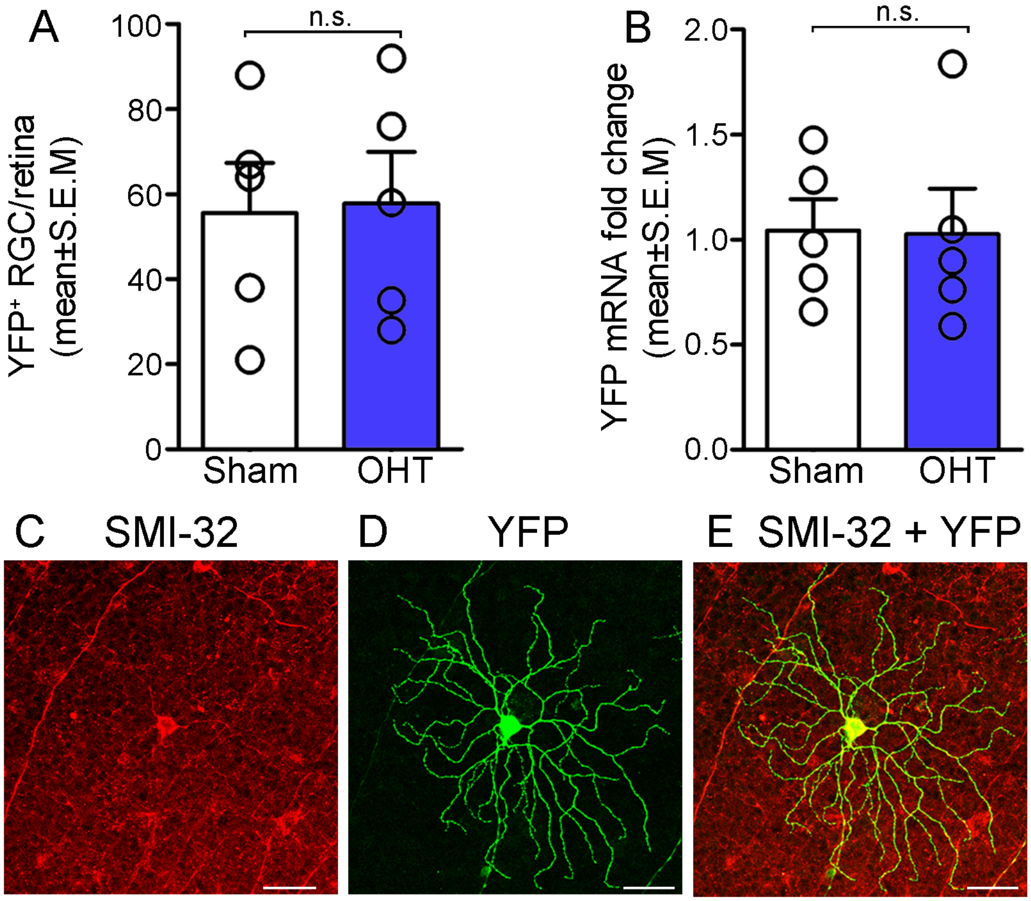

Supplementary Figure 1

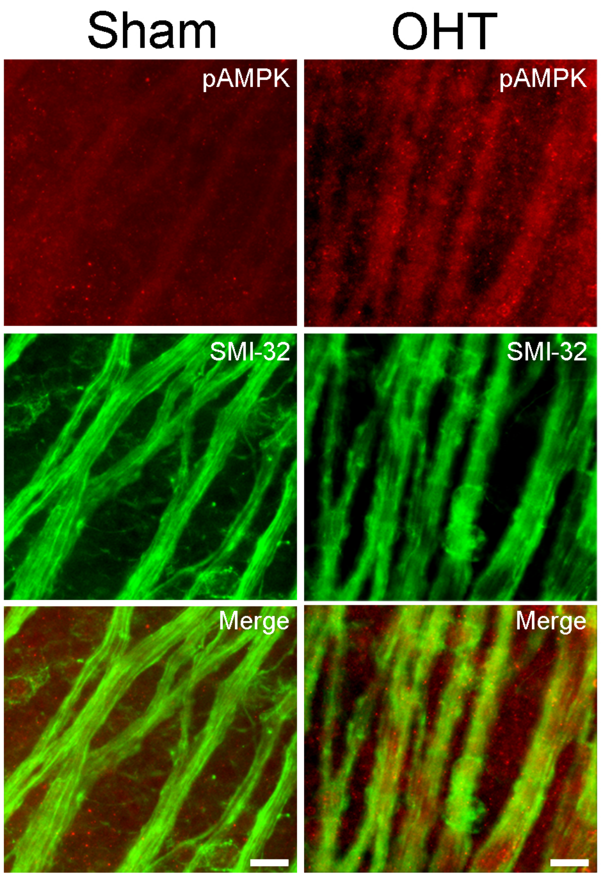

Supplementary Figure 2

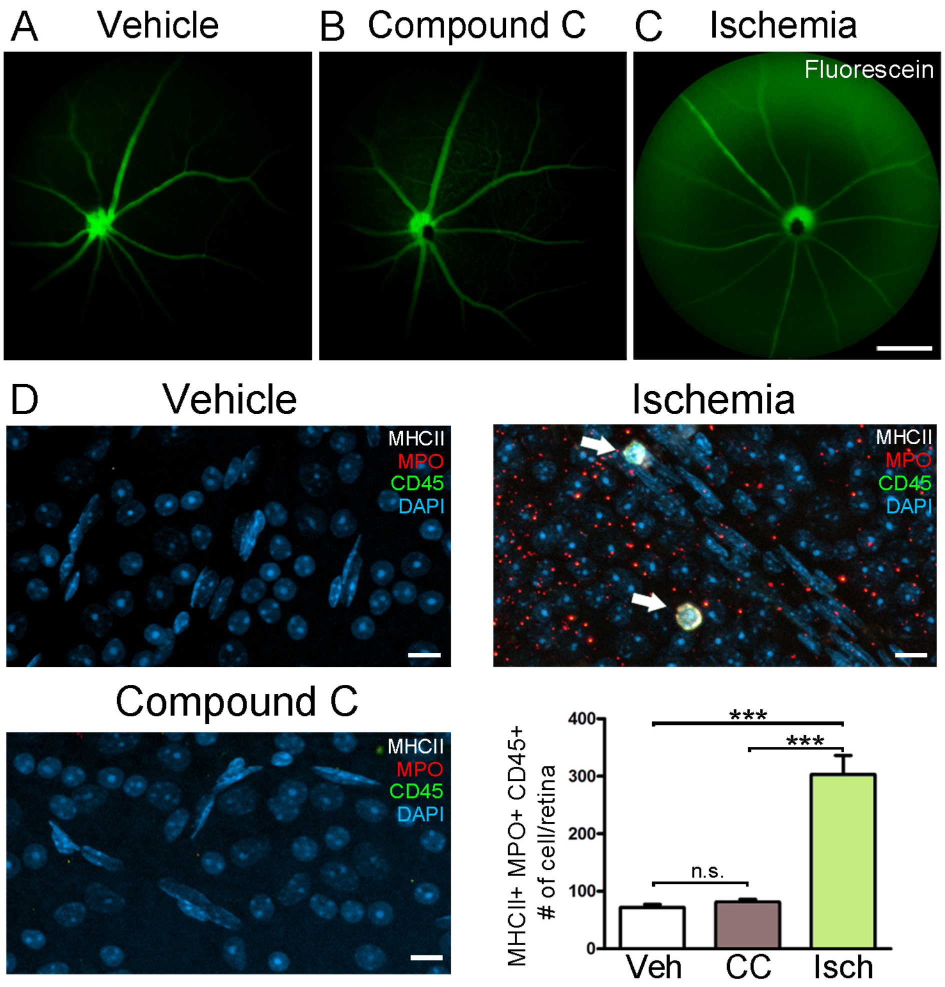

Supplementary Figure 3

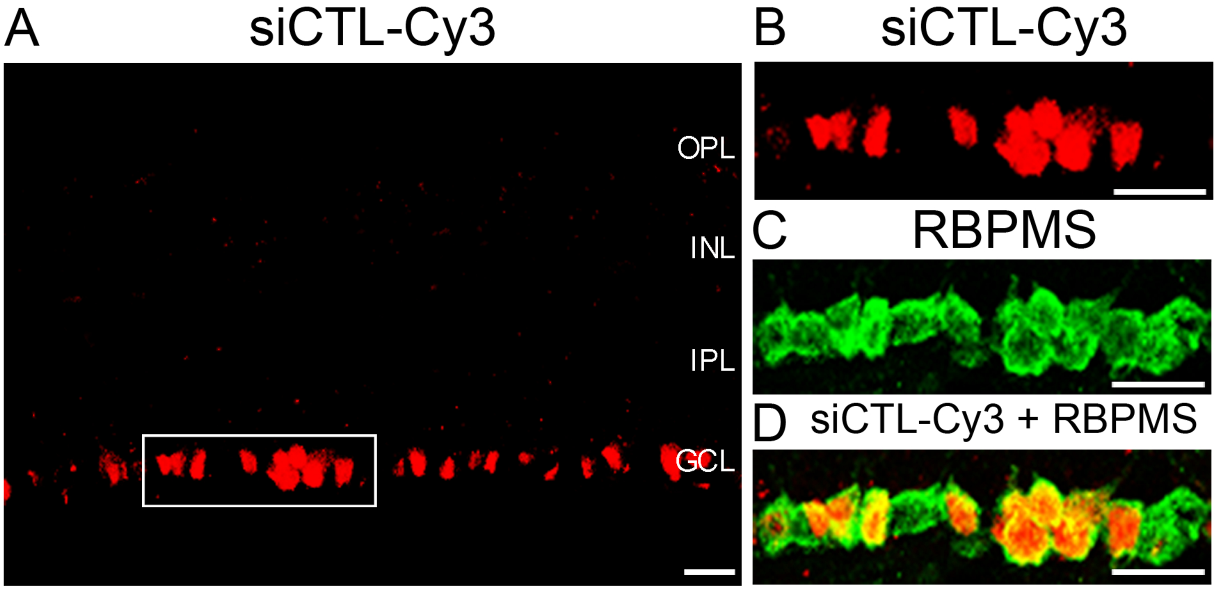

Supplementary Figure 4

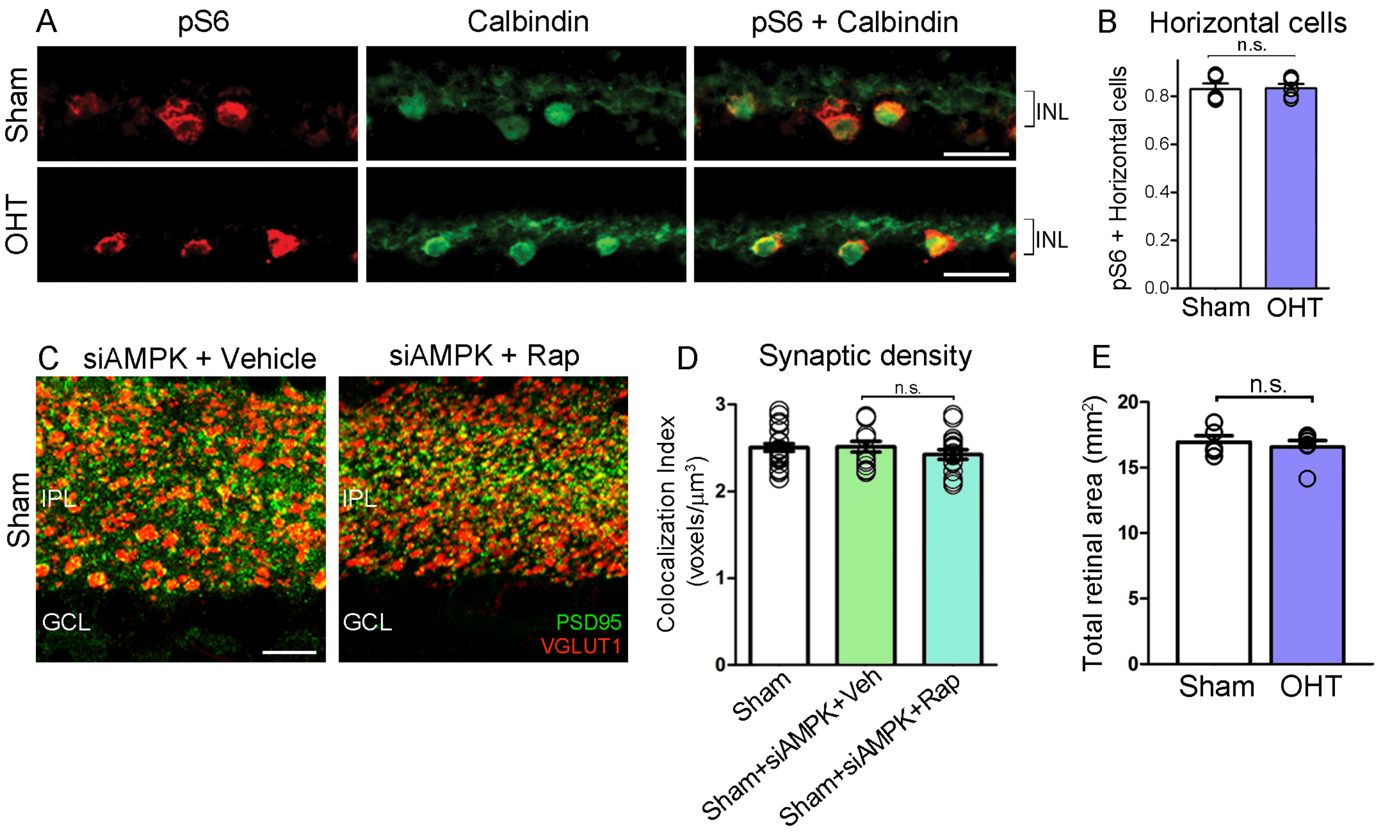

Supplementary Figure 5

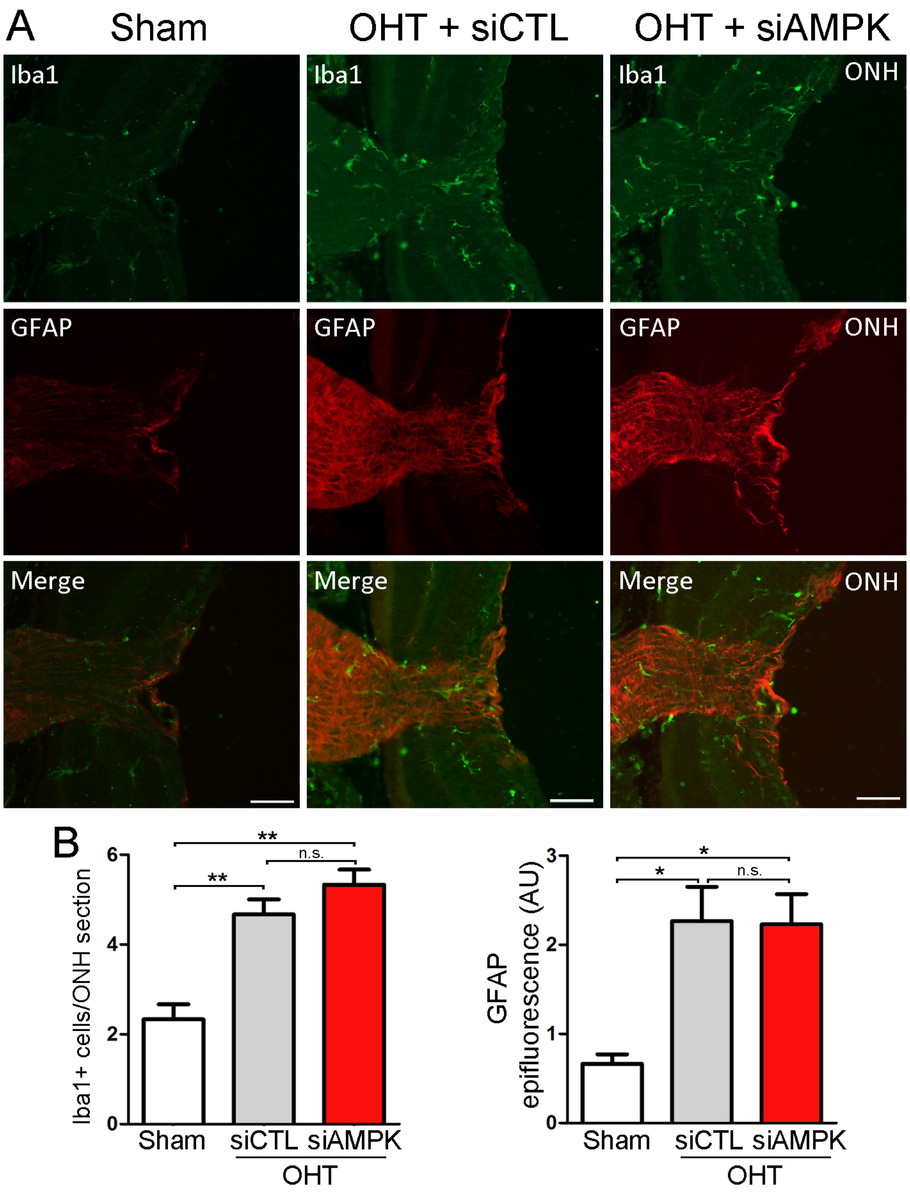

Supplementary Figure 6
